# Intelligent systems for sitting posture monitoring and anomaly detection: an overview

**DOI:** 10.1186/s12984-024-01322-z

**Published:** 2024-02-20

**Authors:** Patrick Vermander, Aitziber Mancisidor, Itziar Cabanes, Nerea Perez

**Affiliations:** Department of Automatic Control and Systems Engineering, Bilbao School of Engineering, University of the Basque Country (UPV/EHU), Plaza Ingeniero Torres Quevedo, 48013 Bilbao, Spain

**Keywords:** Anomaly detection, Assistive technology, Machine learning, Monitoring and diagnosis, Sitting posture, Wheelchair, Overview

## Abstract

The number of people who need to use wheelchair for proper mobility is increasing. The integration of technology into these devices enables the simultaneous and objective assessment of posture, while also facilitating the concurrent monitoring of the functional status of wheelchair users. In this way, both the health personnel and the user can be provided with relevant information for the recovery process. This information can be used to carry out an early adaptation of the rehabilitation of patients, thus allowing to prevent further musculoskeletal problems, as well as risk situations such as ulcers or falls. Thus, a higher quality of life is promoted in affected individuals. As a result, this paper presents an orderly and organized analysis of the existing postural diagnosis systems for detecting sitting anomalies in the literature. This analysis can be divided into two parts that compose such postural diagnosis: on the one hand, the monitoring devices necessary for the collection of postural data and, on the other hand, the techniques used for anomaly detection. These anomaly detection techniques will be explained under two different approaches: the traditional generalized approach followed to date by most works, where anomalies are treated as incorrect postures, and a new individualized approach treating anomalies as changes with respect to the normal sitting pattern. In this way, the advantages, limitations and opportunities of the different techniques are analyzed. The main contribution of this overview paper is to synthesize and organize information, identify trends, and provide a comprehensive understanding of sitting posture diagnosis systems, offering researchers an accessible resource for navigating the current state of knowledge of this particular field.

## Introduction

Every day, the sedentary lifestyle is on the rise among individuals. It is estimated that on average, these people spend more than half of their daily hours in a seated position, reaching up to 85% of their hours in the case of people with low mobility [1]. The cause of this sedentary lifestyle is strongly linked to bone and muscle weakening caused either by aging or a neurodegenerative disease [2].

This muscle weakening causes people with low mobility to make use of an assistive device such as a wheelchair that allows them to move around normally [3]. While using a wheelchair contributes to an improvement in the mobility of its users, thus allowing an increase in their independence, its use is associated with an increase in the sitting posture, bringing about the psychological and physical problems that this involves.

The physical problems of wheelchair users generate an intrinsic inability to carry out an adequate postural behavior [4]. The adoption of a good posture is essential, since the effects of sitting posture anomalies include the appearance of back, shoulder and neck pain, muscle tension and nerve problems, among others [5]. On the other hand, the postural state of a user, understood as the position adopted by the human body at a given moment, can be indicative of its functional state. Thus, the appearance of anomalous postures may be indicative of a change in this functional state. This change may be due to a characteristic worsening of neurodegenerative diseases, such as outbreaks in multiple sclerosis, or an effective recovery from rehabilitation, being a patient recovering from a stroke a clear example [6].

Given the information provided by knowing the postural status of wheelchair users, it is of vital importance to carry out a postural diagnosis. To date, postural diagnosis has been performed exclusively by medical specialists, using both medical observation (visual) and questionnaires to be completed by the patient [7, 8]. However, these methods have a number of limitations. First, the fact that to date it is mainly carried out in clinical settings given the impossibility of continuous follow-up by the health specialist at home, and, in addition, the subjective component characteristic of the patients in the questionnaires to be filled in.

Thus, it is hypothesized that the use of an intelligent postural diagnosis system can be beneficial for the early prevention of changes in the functional status of users, thus being able to adapt rehabilitation to each user and pathology [9]. This adapted rehabilitation can help prevent possible musculoskeletal problems caused by the adoption of undesired postures, which together with the consequences of each pathology worsen the quality of life of the user. In the same way, it can be used to prevent risk situations such as the appearance of ulcers in patients who spend time in the same posture without moving [10]. It is also possible to analyze possible situations of falls due to lack of control and strength over the trunk [11], due to the consequences of the disease itself, such as paralysis of part of the body in the case of stroke patients. In short, by adjusting rehabilitation to the newly identified casuistry, the recovery process is optimized, the risks of complications derived from inadequate postures are reduced and a better quality of life is promoted for affected individuals [12].

**Fig. 1:**
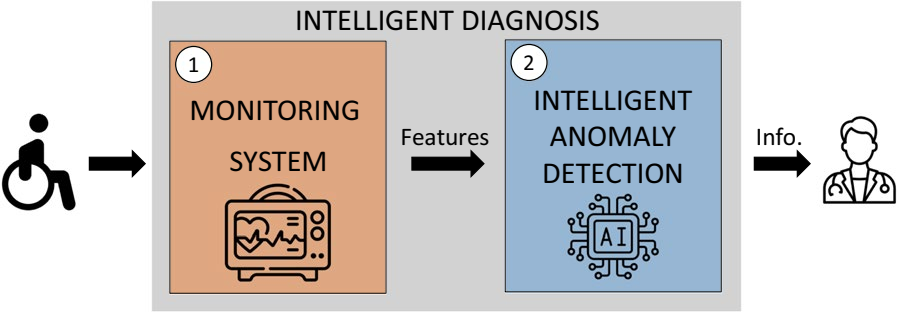
Intelligent sitting posture diagnosis scheme based in two steps: monitoring system and intelligent anomaly detection

These postural diagnosis systems can be divided into two stages (Fig. 1): A first stage in which a postural monitoring is carried out, capturing postural data of interest, and a second stage in which statistical [13–23] or intelligent techniques [5, 15, 17, 19, 24–32], among others, are used to identify the user’s postural state.

Different postural diagnostic systems based on these two stages have been proposed in the literature. However, most of these devices are oriented to a population far from that of wheelchair users, being these works largely focused on office workers [13, 17, 33, 34] or students [35, 36]. Wheelchair users have a number of characteristics that are common to them and different from the rest, and therefore require a particular approach.

As postural diagnosis is based in two steps, monitoring and anomaly detection, it is of interest to make an analysis of the existing devices based on the postural data collection system and another analysis dedicated to sitting posture anomaly detection techniques. While in [37] an analysis of monitoring devices and in [38] a compilation of postural classification techniques is carried out, no work presents a complete analysis of both devices and techniques that explore in depth into the problem of detecting sitting posture anomalies, that is, the whole diagnosis systems are not reviewed. Furthermore, the focus of both articles is distant from wheelchair users, who, as will be discussed later, exhibit distinctive characteristics that must be taken into account when developing such systems.

Therefore, this paper presents a complete analysis of sitting posture monitoring and intelligent anomaly detection systems for wheelchair users. For this purpose, the advantages, limitations, opportunities and challenges of both the devices used for postural data collection and posture anomaly detection techniques subsequently applied to extract knowledge will be presented. In this way, this article works under the assumption that an early sitting posture anomaly detection allows to detect a wheelchair user functional status change. The main objectives of the work are the following:

- Organize information, identify trends and provide a comprehensive understanding of technical monitoring and assistance devices for sitting postural diagnosis.
- Offer reasearchers an accesible tool to navigate the current state of knowledge in sitting posture anomaly detection field, using both statistical and intelligent techniques.

For this purpose, this survey is divided into five well differentiated parts. Firstly, the methodology followed for the bibliographic search is defined in section Materials and methods. The different devices developed to capture postural data in seated position are presented in section Sitting posture monitoring systems. Subsequently, the data processing techniques for the detection of sitting posture anomalies are analyzed in the section Sitting posture anomaly detection techniques. Specifically, this section is divided into two subsections, one dedicated to the analysis of the techniques used to date (section Traditional approach through generalized techniques), and a second one in which the techniques are analyzed based on a new approach (section New and individualized anomaly detection approach). Next, a discussion of the advantages and limitations offered by the different monitoring and analyzed techniques for their application in the postural field in wheelchair users in section Discussion. Finally, the main conclusions of the article and future lines of work are given in section Conclusions.

## Materials and methods

The first step of this analysis involves explaining the search methodology conducted during the literature review process. For this reason, the search process carried out (section Search process), as well as the criteria for inclusion and exclusion of the papers (section Eligibility criteria), are defined below.

### Search process

During the literature search process, a parallel and independent search process has been followed by various researchers. It is important to keep in mind that given the two distinct parts that make up postural diagnosis, this literature search was divided into two, one relating to postural monitoring devices on the one hand, and anomaly detection techniques on the other. This bibliographic search consisted of the following steps:

1. Identification of all works that meet the chosen search criteria. This process is performed within the most common engineering databases such as Web of Science, Scopus and IEEE Xplore. Duplicate works that are repeated across different databases are removed afterwards. For the search, a combination of words belonging to two groups was used. First, the target population was defined. Within this group, words such as: student, worker, wheelchair user, elderly or driver were used. In the next group, the function to be studied was defined with words such as: monitoring, anomaly detection or postural identification. Finally, optional search refinement words are used such as: sitting posture, classification, machine learning, statistics, rules, supervised, semi-supervised, unsupervised, among others. Thus, use is made of the combination of one word from each group with the optional use of one or more words from the refinement group.
2. The second step consisted of screening these papers, filtering out those that are written only in English, as well as those that belong to journal or conference articles. No restriction has been included as to the date of publication of the articles, with the search range being between 2000 and 2023.
3. Of these articles, a first reading is performed, thus making it possible to examine whether the articles that have passed the previous phase meet the eligibility criteria defined below. In the case these criteria are not met, the articles are discarded.
4. Finally, the articles that meet the criteria are read in depth and discussed at this paper. It is in this step where an exhaustive analysis of the works is carried out and the relevant information is extracted from each one of them. It is in this step where the articles selected by each of the researchers in their search process are shared. Those articles selected by both are directly included. The articles selected by a single researcher are discussed, analyzing their inclusion or not by consensus.

### Eligibility criteria

The following is a list of the inclusion and exclusion points that have been taken into account when defining the works to be included in the overview process.

Inclusion criteria:

- Devices of any type that allow the collection of sitting postural data.
- Techniques may include machine learning algorithms, artificial intelligence, statistical methods, or a combination of these to perform anomaly detection.
- Participants with or without musculoskeletal disorders.
- Studies reporting quantitative outcomes related to the accuracy, sensitivity, specificity, or reliability of the sitting posture diagnosis systems.
- Studies conducted in various settings, including office environments, home settings, and clinical or rehabilitation settings.

Exclusion criteria:

- Studies focusing exclusively on participants with medical conditions unrelated to posture.
- Exclude studies conducted on animals.
- Exclude studies relying solely on self-reporting without objective monitoring.
- Exclude studies without relevant posture-related outcome measures.

## Sitting posture monitoring systems

Once the bibliographic criteria have been explained, the discussion proceeds to the results obtained concerning intelligent sitting posture diagnosis systems. To create an intelligent system capable of detecting anomalies in sitting posture, the first step involves designing a monitoring device capable of capturing kinematic and/or dynamic variables, offering insights into the user’s postural status. Among the advantages of carrying out an intelligent postural monitoring is the fact that it allows to know remotely and in real time the postural status of the patient. In addition, the possibility of remote monitoring avoids the continuous presence of specialized health personnel, also allowing monitoring to be carried out outside the clinical environment and thus providing information on the patient’s daily activities. In this way, and following the example of the stroke patient, which will be analyzed throughout this paper, the advantages of carrying out correct postural monitoring is that possible future complications such as relapses in the disease or new strokes can be detected remotely, facilitating early medical attention.

**Fig. 2:**
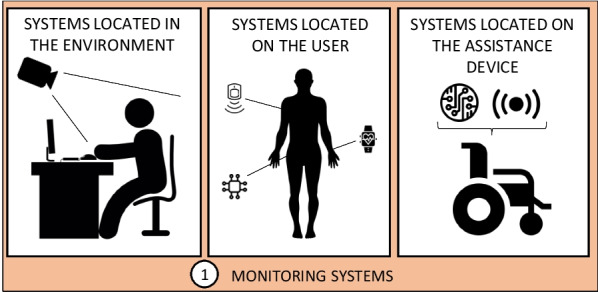
Postural monitoring systems classification based on sensor location

The study of technology in this field allows highlighting different technological solutions for the measurement and monitoring of postural physical variables, being able to place these measuring devices both in the environment and in the user’s own body. In order to analyze the advantages and disadvantages of these devices, it is necessary to pay attention to fundamental aspects for their application in wheelchair users. Among these aspects are the cost and energy autonomy of the device, portability, degree of intrusiveness or adaptability to different wheelchair models. This work proposes a classification based on three large groups, depending on the location in which they are located (Fig. 2): systems located in the environment (section Systems located in the environment), systems located on the user (section Systems located on the user), and systems located on the assistance or support device (section Systems located on the assistance or support device).

### Systems located in the environment

The first set of sensors used for postural monitoring are the so-called ambient sensing systems or systems located in the environment. These systems are based on using one or several sensors located along a limited and delimited surface, thus allowing the measurement of different variables of postural interest. Within this group, the use of devices based on vision or cameras stands out.

Vision systems consist of a set of cameras located in the environment that allow locating reference points on the human body. Among these points, the location of the head, shoulders, arms and hips stand out [33]. Knowing the relative position between these parts of the body, a general idea of the posture adopted by the user can be estimated.

Among the main advantages of using vision cameras for postural monitoring, in addition to the speed of information processing, is the fact that it is a non-intrusive data collection tool [35]. Furthermore, the use of cameras makes it possible to monitor more than one person simultaneously, as long as they are within the cameras’ range of vision. This is of particular relevance in subjects with low mobility such as stroke patients, since the incorporation of technological devices may add an additional degree of restriction in movement, due to, among other things, discomfort from the sensors.

The main limitations of these systems include limited privacy and limited range of action. The monitoring of the user’s posture is limited to the range of vision of the camera. In this way, the moment the user leaves the camera’s field of view, monitoring is interrupted. Thus, applications are limited to stationary environments unless the user is tracked. For this reason, the use of this type of sensors is not widespread for the detection of postural anomalies in motion, as may be the case of wheelchair users. However, the vast majority of works that make use of vision systems for postural monitoring focus on environments where the user is stationary, the most common being office work [13, 17, 33, 34] and student environments [35, 36].

Since the monitoring is performed from a fixed plane, the arrangement of the cameras becomes especially relevant, and more than one camera placed in different perspectives may be needed to achieve a complete monitoring. It is also important to take into account that the location of the sensor will determine the interferences in the form of occlusions between the subject and the camera. Thus, the predominant approach for determining postural anomalies in a seated position involves utilizing a lateral perspective [22, 33, 39]. This perspective allows monitoring the entire human body, as long as the subject does not sit on a seat that obstructs body visibility. However, there are works in which it is proposed to perform the monitoring by placing the camera in a frontal view, monitoring only the upper trunk [17, 34, 36, 40]. Nevertheless, the main objective of these studies is not usually the detection of seated anomalies, but monitoring is used as a means to achieve the ultimate goal of detecting the user’s degree of attention to attend class or work.

However, the main problem of using traditional vision cameras is that the correct processing of the data is conditioned to the acquisition of a clear image by the camera. This image may suffer variations with the environmental conditions, such as luminosity, so it is not considered a suitable measurement system for this type of application. One of the alternatives used by other authors consists in using other types of sensors to monitor people, such as the use of thermal sensors [34] or infrared cameras [33, 40]. While this type of technologies allows to achieve independence between the result of the captured images and the light conditions, it introduces a new limitation, the dependence towards the measured temperature and surfaces. This type of imaging is affected by the temperature at which the sensor is located, giving different measurements indoors or outdoors, and presenting measurement difficulties on reflective surfaces such as water or glass. In addition, infrared technology has limited measurement ranges. Thus, in addition to the existing restriction on the range of action, a new one is added in terms of depth, beyond which measurements are no longer accurate.

**Fig. 3:**
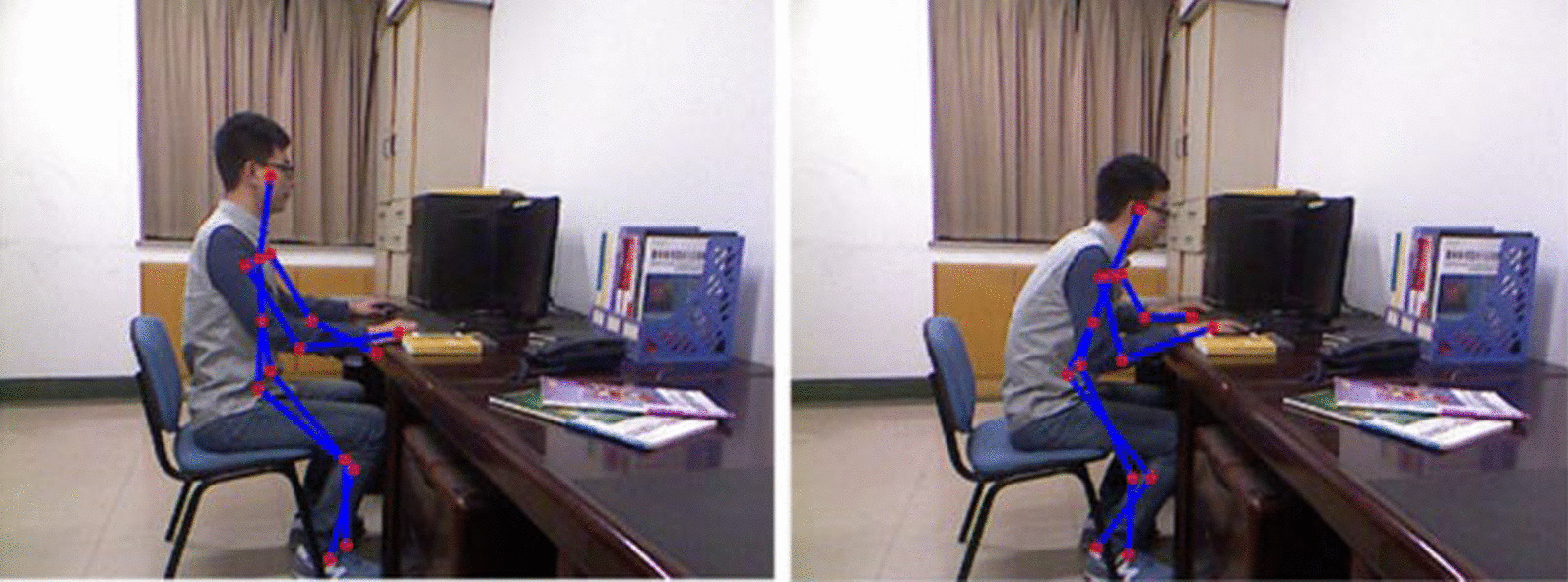
Example of human body modeling using Kinect camera, presented at [13]. On the left, neutral sitting posture. On the right, frontal tilt of the thorax

In order to reach a compromise between traditional vision cameras and those based on infrared technology, depth cameras have been developed. Depth sensors are the technology by which nowadays the postural study is being approached through the use of cameras. Depth sensors allow accurate three-dimensional modeling of the human body by estimating the distance of the points of the body with respect to the camera as shown in Fig. 3. Among the various commercial image capturing devices, Microsoft’s Kinect camera has prevailed over the others. The use of this camera has become widespread in the medical field, being of great use both for postural recognition of users, whether they are sitting users [13, 36] or standing users [22, 41, 42].

In conclusion, this type of technology can have great advantages such as the ability to detect objects and the rapid processing of information. However, it is necessary that they are located in controlled environments, where light conditions remain constant and occlusions between the camera and the subject to be studied are also avoided. That is why they are frequently used to study the correct sitting postures during study hours or work in the office, being not so suitable for people with low mobility, who move for their daily activity in different environments using a wheelchair.

### Systems located on the user

In order to extend the scope of postural monitoring, a second group of sensors located on the user’s own body is proposed. These sensors, commonly referred to as wearable sensors, are a set of sensors incorporated on the clothing or on the body itself and which allow to collect information continuously throughout the day.

These sensors are generally small in size and simple to implement, which is one of their main advantages. Their small size allows the incorporation of the sensors attached to clothing, allowing great portability. This portability is linked to an increase in the degree of invasiveness of the users. Despite their small size, these devices have to be attached to the human body in order to capture data correctly, which makes them uncomfortable. This means that many people prefer not to use them. This discomfort is increased in people with low mobility, with several studies suggesting that this group rejects the use of wearable sensors [24].

**Fig. 4:**
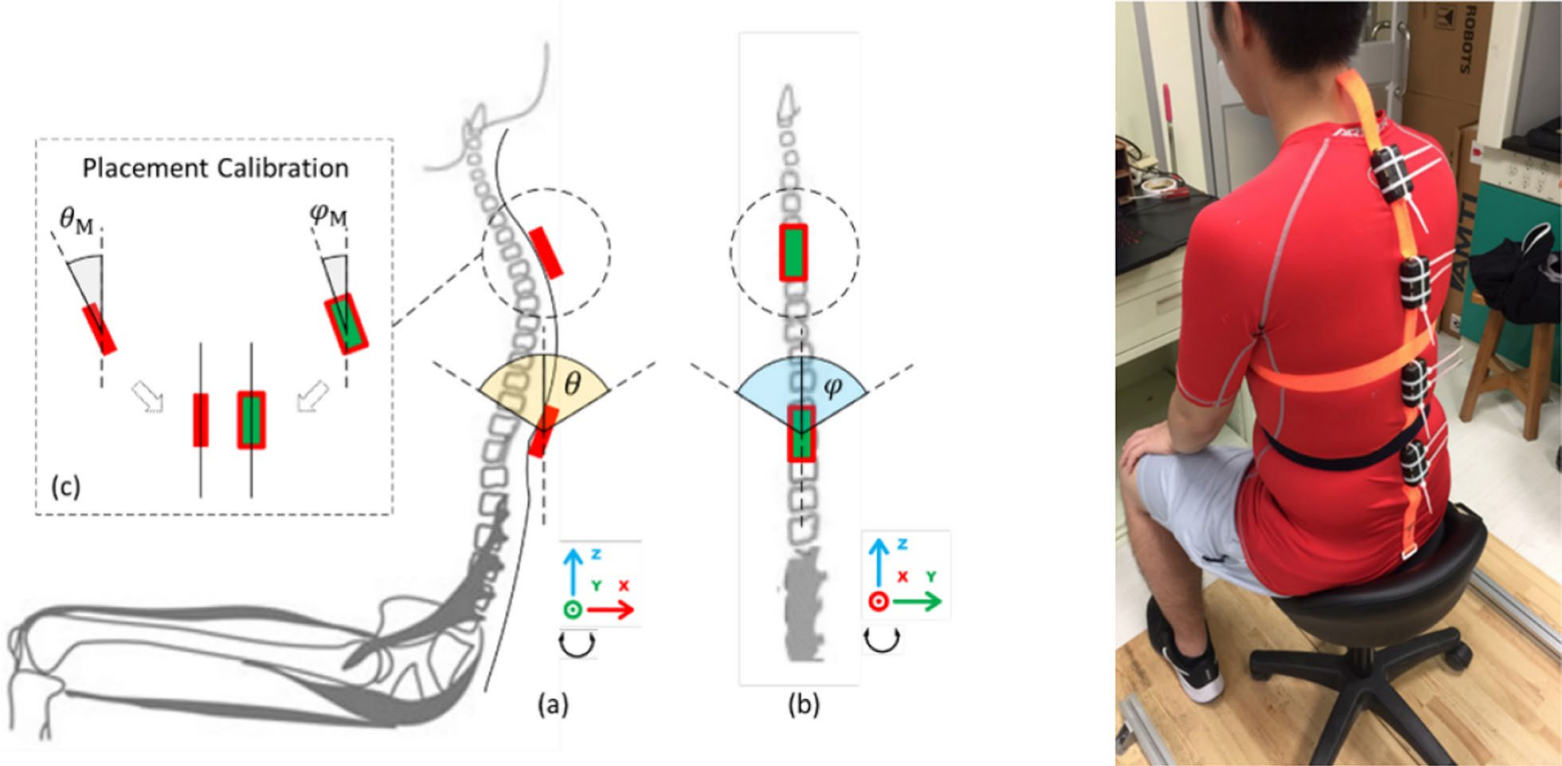
The image on the left shows the location of the inertial sensors between the C7 and L4 vertebrae provided by [49]. On the right is an example of implementation of the sensors on the spine of a test subject of the [45] work

The information obtained from wearable sensors is highly dependent on their location along the human body. Different approaches have been made as to what is the ideal location so that, with the smallest number of sensors a correct monitoring of the spine can be performed. In [43] it is concluded that for a correct estimation of the sitting posture it is sufficient to know the state of the spine between the C7 vertebra and the L4 vertebra, so the sensors should be located along the spine (see Fig. 4). Other frequent locations are the waist, chest and arms. The mode of implementation varies, making use of bands on the waist or torso [14, 44–46], or the design of specific T-shirts for easy incorporation of the sensors [16, 27, 47, 48].

The fact that the location of the sensors is so important for the correct collection of data means that in many cases qualified professionals are needed to adjust the sensors to the patient’s body [48]. In general, wearable sensors often tend to suffer from interference due to contact between the sensor and the clothing or the sensor and the human body, being an additional cause of discomfort and lack of accuracy. Moreover, these types of sensors are more focused on motion capture so they tend to be more used for monitoring dynamic postures such as arm movement when a person is walking or fall detection [50, 51], being not as effective for static posture detection [52], in which a wheelchair user may remain immobile for a prolonged period of time.

Among the different existing wearable sensors, inertial sensors or IMUs are the most widespread for postural monitoring [16, 45, 47, 49]. It is a sensor composed of an accelerometer, a gyroscope and a magnetometer, allowing to know the angular velocities and linear accelerations of those points of the body where it is located, being able to perform a study of the movement. The main use of these sensors is the early detection of falls. The aim is to prevent possible falls both from a recumbent position (mainly in a clinical environment) [51] and from an upright posture [53], being less frequent the detection of falls from a seated posture. Although the main application of these sensors is based on the classification of activities or falls, some studies have made use of them to diagnose the postural state of people in order to be able to apply a treatment or postural correction tool at a later stage [48, 54].

In addition to inertial sensors, the use of other types of sensors that, by themselves or in combination with inertial sensors, allow monitoring of the postural state of users has been studied. Thus, the use of inclinometers has been tested to monitor the spine. These sensors measure the inclination of the plane on which they are placed with respect to the horizontal. In this sense, they have been used to measure the deviation of the spine both in the sagittal plane and in the coronal plane [55]. However, the information provided is insufficient for complete postural monitoring, so it is necessary to use them in combination with other sensors. On the other hand, the possibility of using barometric altimeters has also been studied, which, as the body reclines, estimates the variation in body height by means of the difference in pressures [50]. Nevertheless, the information provided is insufficient for complete postural monitoring. Finally, the use of pressure sensors is contemplated, either in the form of strain gauges [56] or implemented in clothing [27], to monitor the deformations produced in the spine. Despite the potential of this technology, it is still a recent field of study and is therefore not sufficiently developed.

To summarize, wearable sensors allow information to be collected continuously in a simple and low-cost way, but they can be a nuisance for users. In addition, this type of sensor is highly dependent on the location in which it is placed, and is prone to disturbances due to rubbing against clothing or the body. Finally, although they can be of great use in conjunction with other types of sensors, they present difficulties in carrying out correct monitoring on their own.

### Systems located on the assistance or support device

The last group of monitoring systems are based on the placement of the sensors on the support surface where the user sits. In this way, it seeks to combine the benefits of the two previous systems, i.e., it seeks to combine the lack of intrusiveness of the systems located in the environment with the portability of wearable sensors. Within this group, the so-called ’smart chairs’ have been developed, consisting of the placement of pressure sensors along the surface of the seat and/or backrest in order to be able to monitor the distribution of forces adopted by the user.

The most commonly used sensors for postural monitoring are resistive transduction sensors [57], with piezoelectric sensors [58] or capacitive sensors [59] being less frequently used. Among the main advantages of these types of sensors are, on the one hand, their low cost, as well as the possibility of adapting to different sensitivities and pressure ranges depending on the material used. On the other hand, their main limitations are non-linearity problems due to the hysteresis effect and variations due to temperature changes, so they require calibration prior to use.

In the literature, there are two trends in the implementation of pressure sensors: the arrangement of the sensors in the form of a mesh, and the arrangement of the sensors in a discrete unitary form.

#### Pressure mats

The pressure mats are composed of a large number of sensors arranged in a matrix, ranging from 256 sensors for the smallest mats to over 2000 sensors [60–63]. This high number of sensors allows for high resolution pressure mapping. Furthermore, these mats are usually composed of flexible materials and can be adapted to different surfaces. The main disadvantage of these mats is that, being composed of a fixed structure, they have less margin to adapt to the dimensional requirements of the different wheelchair models. In addition, since they are composed of a large number of sensors, the price of these mats is high.

With the increase in the number of sensors used to monitor the pressures exerted by the user, data acquisition and processing becomes more complex, so it is common for the manufacturer to provide software for the proper handling of the information. The features offered by this software usually include sensor calibration, data acquisition and visualization, and tools for further analysis (such as the location of the center of pressures, for example). These tools, however, come with a high purchase price, limited autonomy and the difficulty of storing and accessing raw data by the manufacturer.

#### Force sensors discretely distributed

In an attempt to reduce the cost of monitoring devices and make them more accessible to the general public, while at the same time facilitating access to raw data, the use of unobtrusively located force sensors has increased in recent years. Among these sensors, Force Sensing Resistor (FSR) sensors stand out, which return a voltage difference as a function of the applied force [15, 18, 20, 64–67]. Since it is a sensor that acts as a variable resistor, it does not need a large electronics that accompanies it for its good implementation, being the voltage divider the commonly used circuit. Moreover, as they are discretly distributed, their location can be chosen, thus, allowing to be adapted to any wheelchair dimension.

The location of the sensors is vital for a good capture of the most relevant parts for postural monitoring, in addition to reducing costs and improving the performance and accuracy of the intelligent systems subsequently applied. As these sensors measure pressure in specific areas, and therefore have a limited measurement surface, good positioning of the sensor becomes even more important. Although several studies have been done using this type of sensors, the proper location of the sensors has not yet been defined, with each researcher placing the sensors in a different position. The most common is to locate the sensors both in the seat and in the backrest, thus monitoring a greater number of postures [25, 68, 69]. Similarly, and depending on the chair used, especially in the case of office chairs, it is common to locate the sensors only on the seat [59, 70], at the cost of reducing the number of postures to be monitored. Finally, although being rarer, the possibility of making use of sensors located only on the backrest has been studied [71].

It is important to emphasize that the implementation of the sensors must be done on rigid surfaces to allow a correct reading of the sensors. The seating cushions used by wheelchair users can be of different types (gel, air, etc.), each with different stiffness. Given the variety of cushions, the work carried out does not only lack an exhaustive analysis of the ideal location, but also does not take into account the individual and specific cushion of each user.

**Fig. 5:**
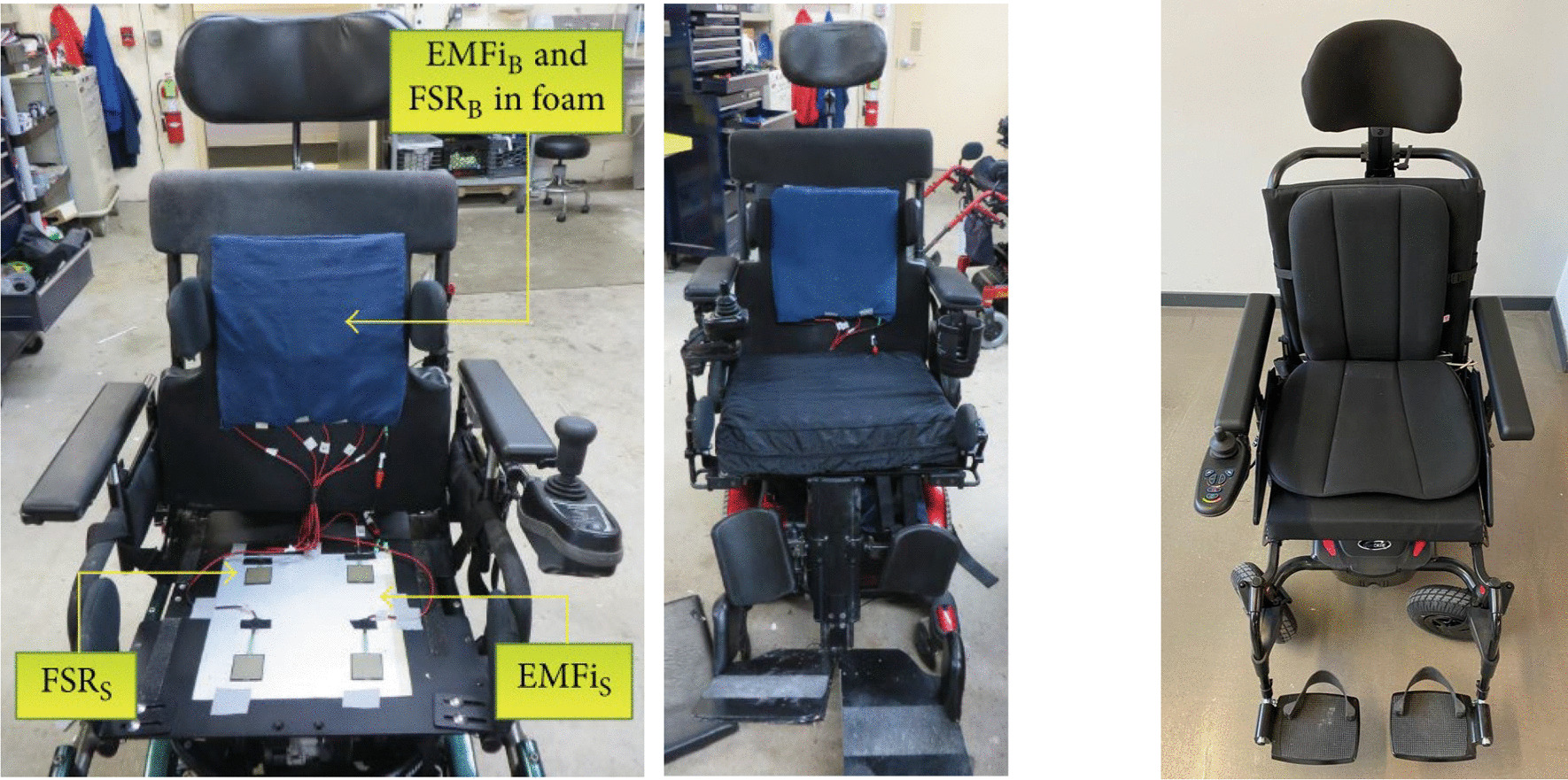
Example of implementation of FSR sensors on an intelligent wheelchair. On the left, example of sensor implementation under the seat cushion [62]. Right, example of implementation of FSR sensors inside the seat cushion [68]

Finally, it should be noted that although pressure or force sensors are the most widely used, the possibility of using other types of sensors located on the support surfaces has been studied. Thus, the use of infrared sensors has been studied which, placed in the backrest, allow the distance at which the back is positioned to be measured, without the need for the back to be in contact with the back [72]. Similarly, the possibility of performing a combined analysis of the postural situation of users and their vital signs has also been analyzed [62] (see Fig. 5). However, all these methods are conditional on the user remaining immobile for prolonged periods, so the transition between postures, and possible anomalies could be missed.

Table 1 shows the classification of the works, with the advantages and limitations of the monitoring devices analyzed.

**Table 1:** Sitting posture monitoring systems summary table

System	Sensors	Advantages	Limitations	Refs.
Environment	RGB	Fast processing speed Not intrusive Simultaneous monitoring	Limited privacy Limited range of action Limited portability	[13, 17, 22, 33–36, 39–42]
	Thermal			
	Infrarred			
	Depth			
User	IMU	Small size Easy implementation High portability	Intrusive Location-dependent Interference due to contact	[14, 16, 27, 43–55]
	Inclinometer			
	Barometer			
	Pressure			
Assistive device	Pressure Mats	High resolution Portability Not intrusive	High purchase price Limited autonomy Raw data accessing difficulty	[60–63]
	FSR	Low cost Portability Not intrusive	Non-linearity Need for calibration Location-dependent	[20, 25, 59, 64–67, 69, 70, 72, 73]

To summarize, among all the monitoring alternatives used to date, pressure or force sensors discretely located on the assistance device offer the best characteristics and are best suited to people with low mobility. It is a non-intrusive solution, which is adapted to the comfort conditions required by this group. In addition, the pressure sensors do not interfere with the postural development of the people they monitor, so measurements are not affected by unusual behavior. This, together with the portability they offer if incorporated into a cushion, makes this type of sensor suitable for postural characterization. However, in order for these sensors to collect information of interest for postural diagnosis, it is necessary that these sensors are placed in a suitable position. Currently, this position has not been defined, and each researcher places the sensors based on different criteria, so the need for further research along these line has been identified.

## Sitting posture anomaly detection techniques

**Fig. 6:**
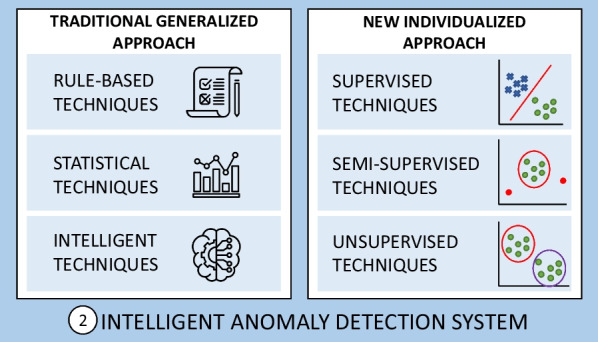
Intelligent techniques for the detection of sitting postural anomalies from a generalist approach

By means of the information extracted from the different postural monitoring methods, it is necessary to quantify and estimate the patient’s postural health status. For this purpose, after a first step of monitoring, it is necessary to carry out a second step of applying anomaly detection techniques to detect abnormal postural states. Different techniques have been proposed in the literature to meet this second step (Fig. 6).

The detection of sitting posture anomalies can be approached from two distinct perspectives (Fig. 6). Traditionally, the normal posture of a user has been considered to be that which involves an upright spine, distributing the weight of the body evenly over the seat and backrest. In other words, the terms correct posture and normal posture have often been used indiscriminately, using a general approach without taking into account the physical conditions of each user. This is due to a large extent to the fact that much of the work is oriented towards maintaining correct postural health in office workers, and not to wheelchair users. Following this definition of normality, abnormal posture is considered to be any posture that is not the correct posture, i.e., upright spine.

However, this traditional approach has a number of limitations. Following the traditional approach, there is a single normality, defined by the correct posture. However, this approach does not take into account the intrinsic particularities of each patient’s pathology, which may result in each user having their own postural pattern. On the other hand, the concept of anomaly has been considered taking the correct posture as a reference, when it is possible that a user, due to muscular weakness, will never be able to perform it. Thus, for example, Thus, for example,people who suffer a stroke usually have paralysis on one side of their body. Because of this, he will tend to lean to one side, and will hardly be able to keep the spine straight. Therefore, under this traditional approach, this person will be under an abnormal postural state constantly.

Therefore, this work considers it interesting to define anomalies based on a new approach. In this approach, the individualized sitting postural pattern is characterized for each user. Subsequently, changes or alterations in these postural patterns that may be indicative that the user’s functional status has changed are sought. These changes in the postural pattern will be considered as postural anomalies. Thus, following the previous example, this person with stroke will characterize his normality by this chest tilt. Under this new approach, any posture that deviates from this lateral tilt will be considered abnormal. Thus, it is possible to detect either a recovery, when he/she manages to keep the back upright, or a worsening, when this tilt becomes more pronounced.

Throughout this section the techniques for the detection of anomalies used to date in the field of postural diagnosis following a classical or generalist approach will be shown (section Traditional approach through generalized techniques), as well as the need to treat the problem from a new individual approach, showing the most common techniques used in other fields (section New and individualized anomaly detection approach). For these techniques, quantitative aspects such as the percentage of success, false positive and false negative rate or computational cost have been analyzed. In addition, other aspects have been taken into account, such as the prior knowledge about anomalies necessary for model training, as well as the effort for data labeling.

### Traditional approach through generalized techniques

As mentioned above, the sitting posture anomaly detection has usually been carried out in a generalist way by detecting all those postures that are not considered to be correct. For this purpose, most techniques have been based on the generation of classification models that allow the classification of different common postures. For this purpose, the techniques used are usually supervised, with the availability of samples labeled as normal and anomalous.

**Fig. 7:**
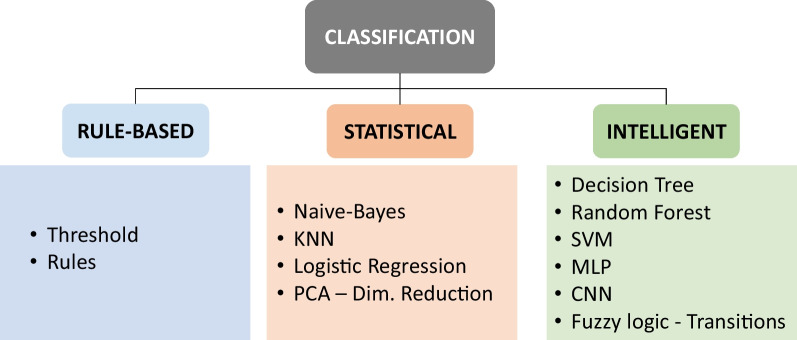
Diagram of the anomaly detection techniques used in the traditional approach

To monitor these anomalies, three types of techniques can be distinguished (Fig. 7): rule-based techniques, statistical techniques and intelligent techniques.

#### Rule-based techniques

The first group of techniques, i.e., rule-based techniques, are based on the assumption that prior knowledge of the postural behavior of users is available. In this way, logical reasoning can be applied to impose the use of rules or syllogisms that allow the postural state of a given user [49, 63, 74].

The main advantage offered by this type of techniques is their simplicity of application. In general, they are based on a low number of rules, being therefore the speed of inference high, allowing them to be implemented in systems with limited computational and time requirements. On the other hand, the classification process is totally transparent and explainable, being formed by logical and understandable rules, thus allowing the user to understand at all times the decision-making process of the model.

Furthermore, this type of rules have a number of shortcomings that may make them unsuitable for detecting sitting posture anomalies. Moreover, as they are designed, expert knowledge is necessary for the definition of the different logical relationships that characterize the postural behavior of the users. In the absence of this expert knowledge, the model no longer has the desired performance. In addition, these techniques have a limitation in terms of complexity. In general, they are not able to capture complex relationships, so the models developed are limited, capturing a limited number of postures, and leaving out the monitoring of postures characteristic of people with low mobility such as thoracic rotation. Finally, these rules can be developed for a specific case, but the developed model does not necessarily have to be generalizable to the rest of the users.

Among the different techniques, using discriminant thresholds is the most common due to its ease of understanding and implementation [63]. This is a widely used algorithm based on establishing a predefined threshold above which two activities or postures can be separated. The characteristics that can be taken into account to perform this discrimination are diverse.

In case of using wearable sensors, the usual approach when using thresholding is to define as a threshold the degree of inclination with respect to the vertical axis [49]. If a certain threshold is exceeded, the posture adopted is considered inappropriate, whereas, if it does not reach that figure, the posture is considered correct. In case the monitoring system is through the use of pressure sensors, the preset threshold is useful to prevent ulcers, locating those points where the specified pressure (the established threshold) is exceeded [63].

In addition to making use of thresholds, these can also be combined with the application of different rules or decisions, thus adding an additional condition to define the postures. Thus, as an example, in [74] it is established that a person is in an upright posture, when the x-axis data taken from an accelerometer is zero, and the acceleration on the z-axis is coincident with gravity. In sedentary sitting, a stepwise binary discrimination process is followed. At each stage, one passes to a subsequent stage or not on the basis of prior verification of the fulfillment of a certain rule. In this way, up to 6 different postures are differentiated [65].

In general, rule-based techniques are computationally inexpensive but lack sufficient discrimination to distinguish postures with a high degree of complexity. These complex postures precisely define the postural behavior of individuals with low mobility. Thus, this type of techniques are restricted for use in binary classification applications, where the result is an all or nothing, i.e., the correct posture or not. For this reason, different sets of techniques that allow a more complex study of postural anomalies are addressed in the literature.

#### Statistical techniques

The second group of techniques is composed of the so-called statistical techniques [13–23]. This set of techniques is characterized by the use of statistical and probability methods to study whether a new sample belongs to a certain class or another, based on the relationships and patterns found in the training data.

Among the main advantages of this set of techniques is, as in the previous case, the interpretability of the results. As with rule-based techniques, a process of understanding the decision-making process of the model can be carried out, thus being able to understand the logical reasoning followed by the model. Not only that, statistical models usually provide probability estimates for each classification class, so it is also possible to know the degree of confidence of the prediction. The possibility of using them in multiclass problems, added to a reasonable training time of the models, makes that this type of techniques has been frequently studied in the literature.

The main limitations of this type of techniques are that they work under different assumptions. Among them, that the training (and new) data follow a statistical distribution, which is the one that allows the estimation to be performed later. Knowing the distribution that the data follow (if they do) again requires knowledge of the database by an expert. Another assumption that is often made is the independence of the input variables. This independence does not need to be true, especially in the face of an increase in the dimensionality of the problem. These techniques are not able to capture the relative importance of the input features and may be sensitive to irrelevant ones. This results in limited performance if the data are complex.

Among the classifiers based on statistical techniques, the Naive-Bayes classifier or Naive Bayesian classifier is in first place [13, 14]. This classifier is characterized by using Bayes Theorem to calculate probabilities. This theorem assumes that the features of a class or object are independent of each other, and therefore, each of them contributes independently to the probability of one class or another. Among the advantages of this type of methods is their simplicity and the small number of parameters required for their implementation. On the contrary, it assumes an independence of variables that in practice is not necessarily true, which can lead to a decrease in the precision obtained. This model has proved to be successful when the number of monitored postures is small [14], however, the results are affected when the number of postures increases significantly [28].

The use of K-nearest neighbor (KNN)-based classification systems have also been proposed in the literature [15–19]. This classifier is based on the construction of a multidimensional feature space where it is assumed that those data belonging to the same class have similar characteristics, and are therefore grouped into nearby clusters. Thus, once the classifier is trained and the feature space is created, to determine the class of a new data, the classes of the K-nearest neighbors are checked. The class to which a larger number of neighbors belong will be the one that is finally assigned. It is a simple algorithm, but it returns high effectiveness ranges. Thus, it has been used in [20] to classify postures that involve a back movement such as tilt or rotation, considered as anomalous. Likewise, in [21] KNN is used for the classification of different postures that involve a change in both the back and legs, mainly oriented to the detection of the postural state in office workers. Nevertheless, since it relies on classifying a posture based on proximity to others in the database, it is necessary to have a diverse and balanced database for all classes. Otherwise, the algorithm will tend to be biased towards those postures that are more frequent in the database. However, a priori, the postures that are not correct and therefore, anomalous based on this approach, will be the most frequent, since they will be all those that are not considered correct. This is why it is not always possible to meet this objective of having a balanced database. Moreover, it is highly dependent on the size of the database, since it has to measure the distance between the new point and all the existing data. Therefore, although it is a technique that does not return bad results, it can be inefficient for high dimensional databases.

Another algorithm used for the determination of sitting posture anomalies based on statistical methods is logistic regression [15, 18]. Logistic regression is based on predicting the probability that a point belongs to one of two mutually exclusive categories. In the postural domain, it is calculated whether a point belongs to a particular posture or not. For this, a logistic function whose coefficients are adjusted during the training phase of the system is used. To carry out a multiclass regression, a logistic probability is calculated independently for each postural class independently and the new data is assigned to the one that returns a higher probability, i.e. to the posture that it is more likely to be. One of the main advantages of this method is that, unlike other statistical methods, it makes no prior assumptions about the distribution of the data, and is capable of handling unbalanced training data sets. Nonetheless, this method does assume that there is a linearity relationship between the different variables, so nonlinear problems are not suitable for this technique. Likewise, it has problems extracting patterns from complex, high-dimensional data, giving lower hit results.

Finally, although it is not a classification technique itself, the use of the principal component analysis (PCA) technique as a preliminary step to the use of other algorithms should also be highlighted [22, 23]. In general, when collecting measurement data, more information is get than is necessary for subsequent posture classification. In addition, many of the features or variables are correlated with each other, resulting in redundancy in the data. As has been shown, one of the main limitations of statistical models is that they have difficulty in dealing with the redundancy of the data and the high dimensionality of the data. Principal component analysis is an unsupervised dimensionality reduction technique that seeks to simplify the input data to a classification model used later. This algorithm does not serve by itself to make postural diagnoses, but as a preliminary step and in order to facilitate the use of a subsequent classifier.

#### Intelligent techniques

The last of the groups of anomaly detection techniques used to date are intelligent techniques or those based on using artificial intelligence. Specifically, Machine Learning techniques are used, a subfield of artificial intelligence that gives devices the ability to ‘learn’ without being explicitly programmed for this purpose.

Among the main advantages of this type of techniques is the fact that they are able to capture complex relationships in the input data, thus allowing more accurate anomaly detection. In addition, these techniques can generalize patterns from training data and apply these patterns to unseen data. On the other hand, they are more adaptable to changes in the input data, being able to retrain the model without the need for large adjustments. Finally, they can detect correlations in the input data, often not evident to humans, and can automatically learn the relative importance of the input features, eliminating or ignoring those that are redundant [75].

Among the main disadvantages of this set of techniques is the need for large amounts of data compared to previous techniques in order to characterize the existing patterns in the data. This increase in the number of training data is directly related to an increase in training time, as well as in the computational resources required to carry it out [76]. The difficulty of choosing the hyperparameters of the models requires a more complex adjustment and configuration than the previous techniques, requiring expert knowledge to do so. Finally, unlike the previous models, this set of techniques usually presents problems when interpreting the results, commonly referred to as black box models, in which the decision-making process is unknown to the user [77, 78].

One of the techniques used in the field of bioengineering for the determination of anomalies are hierarchical methods. These algorithms are based on decision-making, where the responses lead to different nodes where a new decision-making process occurs. This continuous decision making procedure is continued until a point is reached where the answer to the decision turns out to be one of the searched classes. The decision trees [5, 15, 17, 24] is an algorithm that allows to automate this successive decision making process. In this way, the algorithm decides, by means of a previous training in which an analysis of the input data and classification classes is performed, which is the tree configuration that maximizes the probabilities of performing a successful classification. Among the major advantages of using this technique is the fact that it is computationally efficient and does not require prior scaling of the data, saving additional data preprocessing time. In addition, this set of techniques allows the identification of those features that are most relevant for decision making, gaining in interpretability of the results. However, they may have limitations in case the relationships between the different postural variables are on a global scale, since the decision making is based on local divisions of the features. Moreover, to achieve high accuracies, the number of postures to be classified is highly limited. This is reflected in [5], where decision trees are used to differentiate whether a person is sitting, lying or walking. Although high accuracies (98.6%) are achieved, they are limited to monitoring only three actions. Similarly, in [24] decision trees are used for fall detection.

Another hierarchical method used is the Random Forest technique [25–28]. This is a variant of decision trees. While in decision trees, the entire feature set is used during training to build the model, in Random Forest, a part of the global feature set is used to build a decision tree and obtain a classification result. This process is repeated, creating different trees, each consisting of a different data set, and obtaining a classified class from each of them. The class that is most repeated among the responses of the different trees is the result returned by the Random Forest algorithm. This procedure allows to achieve greater precision, as well as a greater capacity for generalization. As in the previous cases, despite achieving high percentages of precision in the model (over 90%), the number of positions is still limited (maximum of 7). All this, at the cost of losing interpretability in the results, which can be vital in order to be able to provide relevant postural information to health specialists. In addition, it requires a higher computational and memory cost, so it may not be suitable for systems with limited resources.

Another technique to highlight is the Support Vector Machines (SVM) [15, 19, 29–32]. The SVM algorithm is a supervised method capable of classifying samples through the use of a separator. While this technique falls under the category of linear separators, it’s important to note that the separation between classes doesn’t necessarily have to be linear. The algorithm of this classifier is based on the search for a separation hyperplane that is equidistant from the closest points of each class. It is also sought that there is the maximum possible margin between classes. Once the model has been trained and the optimal hyperplane has been found, the position of the new data with respect to it is evaluated to decide to which class it belongs. In general, this technique is widely used in biomedical applications, since it is effective for small data sets. In addition, it has a high generalization capability. However, it can give problems in high dimensionality data, especially in those problems with many classes. This is especially detrimental in the case where the number of postures to be studied is high. Thus, in [30], despite the fact that a high number of postures can be classified, studying a total of 12, the accuracy of the classifier is reduced (hit results below 80%). Similarly, in [29], four load cells are used for the classification of 6 common postures. However, both works are oriented to office workers. This population presents a different postural problem from that of people with low mobility, with postures different from those of wheelchair users, such as displacements on the seat or leg movements.

Finally, techniques based on neural networks are used, which have proven to be one of the classification techniques with the greatest potential in the field of bioengineering applications [79], having grown considerably in recent years. The network is composed of layers formed by interconnected layers, where each neuron transmits the input information modified by a function of its own and multiplied by the specific weight to the successive layers. In this way, a structure with the capacity to process large amounts of information and to identify trends and classify postures based on them is achieved. This system is based on automatic learning by means of a previous training, where the different specific weights of each of the neurons are weighted.

There are several typologies of neural networks, which are classified according to the structure of the layers or the interneuronal connections, among other aspects. One of the most widely used neural network models are the Multilayer FeedFordward or Multilayer Perceptron (MLP) networks [66, 68, 80–82]. This type of networks are characterized by having an input layer and an output layer, together with an undetermined number of hidden layers. Each of the neurons in one layer is interconnected with all the neurons in the next layer. This means that the number of parameters in this type of network can reach very high levels. This is a supervised learning method that uses the Back-Propagation algorithm for training. This algorithm, based on the gradient descent method, seeks to modify the weights of each neuron to reduce the global error, starting from the last layer and continuing with the preceding layers. Thus, in [75, 80, 81], use this type of networks are used to perform recognition of activities, such as climbing stairs, or running, as well as various postures. However, these works focus more on activity recognition and do not put their focus on postural diagnosis in seated position of wheelchair users.

Another type of networks used for postural classification are convolutional neural networks (CNN) [76–78, 83]. This type of networks, first perform a process of extraction and reduction of the dimensionality of the features and then go on to perform the classification based on these features. These networks are focused on the field of artificial vision and image processing. Thus, they are used with data extracted using depth cameras for anthropometric scanning of the human body [76], as well as with data extracted using pressure mats for postural diagnosis [77, 78]. After all, as explained in [83], pressure distribution signals extracted from pressure mats can be treated as images. Nevertheless, there are small variations that differentiate it from images extracted through cameras. Among these differences is the fact that the sensors are not isolated and therefore the pixels depend on the force exerted on the surroundings. However, these studies, despite achieving superior performance with respect to previous methods, also in part because the input data set is larger, the computational requirements grow in the same way. In addition, they require a large number of input data for the model to be effective, and they have difficulty in detecting small details within the overall image. Therefore, they can present problems in detecting certain anomalies, especially if they are similar to another set of studied postures.

Most classifiers used in the literature focus on the classification of static postures and activities, which require a period of time for the data to stabilize. However, transitional states between different postures can be a challenge when detecting anomalous postures, since they can be mixed. That is why, some authors [84, 85] decide to make use of fuzzy logic techniques for the optimization of transient or switching postures. Fuzzy logic arises from the idea of emulating human thinking, where sometimes not everything is true or false, and there are middle terms whose degree of definition is often imprecise. In this way, and derived to data processing, fuzzy logic aims to emulate a decision-making tool where the input information is imprecise or ambiguous. The features are described by means of membership functions, which are subjected to rules to infer an output class. Thus, although fuzzy logic is not used as the sole method of diagnosis, the use of these techniques allows the increase of the percentage of success in combination with other techniques, by eliminating the existing inaccuracies in the diagnosis between positions, or transition positions.

In conclusion, postural classifiers has been widely used in the literature for the detection of postural anomalies following a traditional generalist approach. Techniques for the development of these classifiers can be classified into rule-based, statistical and intelligent techniques. While intelligent techniques have high performance and the ability to classify a larger number of postures, they have the counterpart of increased computational requirements and lack of interpretability of decision making. This is why other authors prefer to make use of statistical or rule-based techniques, based on expert knowledge of the database. The complete set of advantages and disadvantages, as well as the above cited references are summarized in Table 2.

**Table 2:** Summary table of anomaly detection techniques with the traditional approach

Techniques	Advantages	Limitations	Refs.
Rule-based	Application simplicity High inference speed Low computational cost Transparent and explainable process	Expert knowledge required Difficulty in capturing complex relationships Limited to a low number of postures and differentiated from each other	[49, 63, 65, 74]
Statistical	Interpretability of results They provide probability estimations for each class Reasonable training time	Assumption that the data follow a probability distribution Expert knowledge required Sensitive to irrelevant features Limited performance with complex data	[13–23, 28]
Intelligent	Ability to capture complex relationships Greater accuracy Adaptable to input changes Ability to ignore redundant features	Need for large data sets Increased training time and computational resources Difficulty in selecting hyperparameters Lack of interpretability of the decision making process	[5, 15, 17, 19, 24–32, 66, 68, 72, 75–85]

Thus, it can be seen how the works that make use of techniques based on machine learning stand out above the rest due to the advantages they offer. However, there are still shortcomings in these works to be taken into account as future lines of research. Firstly, it should be noted that the classification techniques used to date are mainly oriented to a population different from people with low mobility. For this reason, there is a lack of works developed in clinical settings as well as an approach from health perspective to detect wheelchair common sitting postures.

Moreover, this approach is based on the generalizability of classification models. Considering that the wheelchair population is very varied in physical complexions, it is necessary that the analysis around these models further deepen this aspect. The works achieved so far generally yield good results, but they are not typically analyzed in individuals with diverse physical builds. Therefore, it is necessary to study more extensively how the results may be affected for the diverse population.

### New and individualized anomaly detection approach

The vast majority of studies address postural anomaly detection as a common problem for all users, without taking into account the pathology of each patient. However, each user exhibits a unique and characteristic postural pattern. Within the same pathology, different individuals may display varying behaviors based on their physical characteristics [86]. Similarly, the normality for each individual may not be the same, and therefore, a general solution cannot be applied.

Therefore, as mentioned earlier, in a new approach, the goal is to characterize the individualized sitting postural pattern for each user. Subsequently, changes or alterations in these postural patterns are sought, which may be indicative that the user’s functional state has changed and may require a new healthcare intervention.

However, there are few studies that consider this approach in the field of postural diagnosis. Thus, there are works that consider all situations outside the users’ usual behavior as anomalous, such as anomalous activities of daily living, as seen in [87–89], or falls [90, 91]. In the the postural field, as mentioned earlier, postures that do not correspond to the correct postural state are considered anomalous. However, to date, there are few studies that address the detection of postural anomalies in a seated position, defined as deviations from a normal pattern [92].

That is why, to detect changes in the normal sitting pattern, understood as the set of postural states that occur in a subject over time, it is necessary to study the techniques used to solve problems with similar characteristics in other fields. Changes in this pattern can occur in two distinct ways. On the one hand, the appearance of a postural state different from those that constitutes the sitting postural pattern can be considered anomalous, treating it as an isolated and specific case. On the other hand, a change in the sequence in which the postural states of the user’s postural pattern occur can be considered anomalous, treating it as sequential or contextual anomaly. Regardless of the type of anomaly, techniques for detection can be classified into three main groups based on the learning method employed. Thus, these techniques can be classified as supervised, semi-supervised, and unsupervised techniques.

#### Supervised techniques

**Fig. 8:**
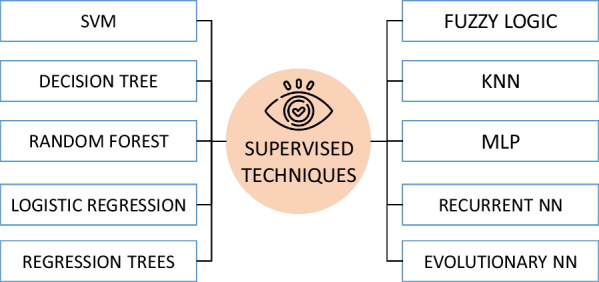
Supervised techniques for anomaly detection from a new individual approach

Supervised learning techniques are characterized by having labeled data representing normal and anomalous classes for training. In this way, through training on the data, a boundary can be drawn to separate the classes defined as normal from those defined as anomalous. It is important to note that throughout this section, studies conducted in different application domains than that of seated posture are presented. Therefore, within this category, techniques based on classification are prevalent. These are similar to the techniques described in the previous section and shown in Fig. 8, so a brief overview will be provided.

Among supervised machine learning techniques for anomaly detection, the use of Support Vector Machines (SVM) stands out, achieving classification accuracy rates exceeding 90% [93, 94]. Other authors prefer using anomaly classification techniques based on decision trees. Standard Decision Trees (DT) [95] and Random Forest [93, 96] are employed in this context. Additionally, some studies utilize regression techniques, including linear regression [97], logistic regression [95], and regression trees [96].

There are other studies that employ common classification techniques, although these are not as prevalent in anomaly detection. Specifically, fuzzy logic is utilized in some works [34, 98], as well as the k-Nearest Neighbors method [99]. Lastly, attempts have also been made to use techniques based on deep learning. In particular, multilayer perceptron neural networks have been employed [100], recurrent neural networks [101] for handling temporal or sequential data, and evolutionary neural networks [102] that automatically adjust the network structure based on evolutionary algorithms.

In general, all these studies achieve high accuracy rates in detecting anomalies in domains such as intrusion detection or network anomalies, as they are based on the assumption that labeled data is available for both normal and anomalous classes. However, in reality, this is highly complex, as typically, the number of anomalous samples is much lower than the number of normal samples. This leads to imbalanced databases, favoring the detection of normal classes over anomalous classes.

For this reason, supervised learning techniques are generally not widely used in anomaly detection in the medical field and are relegated to very specific cases. This is mainly due to the fact, on the one hand, that the number of anomalous classes is lower than the normal ones, and on the other hand, that the presence of a health specialist is necessary for the labeling of these samples. To address the challenges inherent in supervised techniques, many authors have opted for the use of semi-supervised techniques.

#### Semi-supervised techniques

Anomaly detection techniques based on semi-supervised learning operate under the assumption that only samples labeled as normal are available. The underlying principle of these supervised techniques involves learning to characterize the normal class through a training process using normal samples. A new data point entering the system is considered anomalous if it does not belong to the normal class defined during this training.

**Fig. 9:**
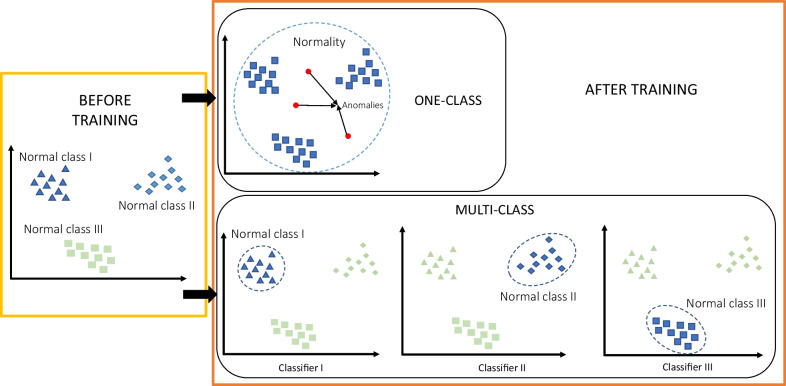
Scheme for the use of one-class and multiclass semi-supervised techniques

Within this section, classification techniques adapted to the availability of only normal class samples are also employed. Two different approaches can be distinguished within these techniques, represented in Fig. 9. On one hand, there is one-class classification, which assumes that all normal samples belong to a single class labeled as the normal class, and anything outside of this class is classified as anomalous. Notably, within this category, techniques based on one-class SVM stand out [103–105]. The main issue with this approach arises when the normal classes forming the data are very different from each other, resulting in a broad boundary characterizing normality. As a result, the occurrence of an anomaly is more likely to fall within that boundary, leading to a false negative.

Hence, other studies shift towards a multiclass approach [106]. This approach is very similar to the previous one, but instead of having a single normal class, a one-class classifier is built for each normal class. When a new data point arrives, it is determined with each trained classifier whether the sample belongs to any of the defined normal classes. If it does not belong to any, the sample is considered anomalous. The primary challenge with multiclass classification methods lies in the need for different labels for each of the normal classes. This once again necessitates the involvement of healthcare professionals to provide such labeling.

To eliminate the need for labeling different normal classes, distance-based techniques are employed. Among various methods, a commonly used technique is k-Nearest Neighbors (KNN). These methods operate on the assumption that an anomalous data point will be distant from the rest of the dataset. For instance, in [107], a variant of KNN (TCM-KNN) is presented where the distance is weighted for each point, and in [108], a modified method (CSI-KNN) takes into account the strangeness and isolation for each point to weigh the degree of anomaly. The issue with these techniques lies in the fact that if anomalous data points are close to a set of normal data, they may be characterized as normal, leading to worse results.

In addition to classification and distance-based techniques, other methods have been employed to determine if a new sample deviates from the known normality. These models are based on the assumption that the data follows a probabilistic pattern. Thus, during the training phase, this postural pattern is characterized, and a new sample is considered anomalous if it deviates significantly from the pattern. Probabilistic techniques based on Gaussian models have been used for point anomaly detection [19, 109], and Hidden Markov Models (HMM) have been applied for sequential anomalies [67, 110]. The challenge with these models lies in the fact that if the data does not conform to a statistical model, the results may not be satisfactory.

There are also studies where deep learning techniques are employed for anomaly detection. The use of multilayer perceptron neural networks is prevalent [111, 112], or deep belief networks (DBN) [113] in two phases, an initial unsupervised phase and a subsequent one for fine-tuning the parameters. In the training process of these networks, the goal is to enable them to distinguish between different existing normal classes, providing a confidence level for each inference. However, when faced with an anomaly, the network struggles to assign it to any of the established classes with the required level of confidence. Similarly, for the detection of sequential anomalies, recurrent neural networks and Long Short-Term Memory (LSTM) networks are employed, where disruptions in the temporal or discrete sequences constituting a defined pattern are detected [112, 114].

**Fig. 10:**
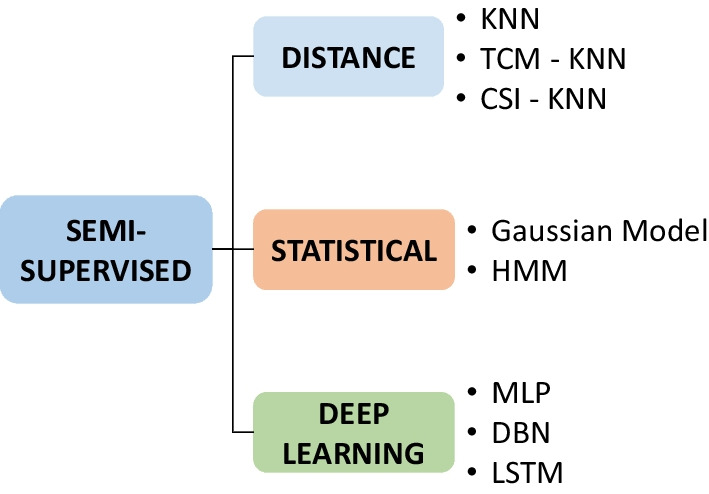
Semi-supervised techniques for anomaly detection from a new individual approach

In general, semi-supervised methods (see Fig. 10) have been commonly used for anomaly detection in various domains, always understanding anomalies as deviations from a pattern of normality. Working under the assumption that only normal data is available, the concept of an imbalanced dataset during training becomes less critical. However, having a known database where no anomalous data is present is essential, or the training process could be adversely affected. In sitting posture anomaly detection, since the postural pattern is composed of several postural states, the presence of a health specialist is once again necessary to allow the labeling of the different normal classes.

#### Unsupervised techniques

Finally, in order to try to solve the main limitations of semi-supervised techniques in the field of postural monitoring, unsupervised techniques could be used. These are the most commonly used techniques when detecting anomalies in different fields [115]. Unsupervised techniques are characterized by not having the label or class of the data in the database, and work under the assumption that data belonging to normal classes are grouped into clusters, with anomalies being far from these clusters. In case this assumption is not met, unsupervised techniques suffer from a high number of false positives [116].

**Fig. 11:**
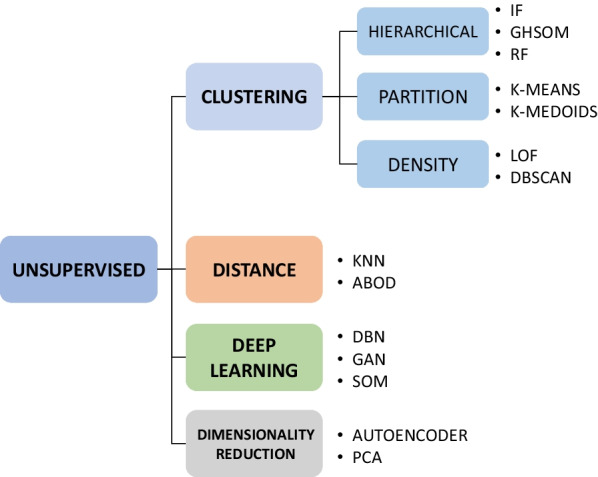
Diagram of the unsupervised techniques used for anomaly detection with a new individual approach

Unsupervised anomaly detection algorithms can be classified in different ways, as seen in Fig. 11. One of the most commonly used techniques is clustering. Clustering is the set of techniques that aims to group samples with similar characteristics into different groups (or clusters), such that each group presents a common behavior or pattern. Clustering techniques can be divided into three types: hierarchical clustering methods, partitional clustering methods and density based methods.

Hierarchical clustering methods allow a hierarchical grouping of clusters. In this way, similar data are grouped into clusters. These clusters in turn are grouped together to form larger clusters. In this way, a hierarchical tree structure is created, where at each level the data is arranged in a different number of clusters. Thus, for the detection of anomalies, Isolation Forests (IF) have been used on the one hand [117, 118]. By means of these forests, the initial data set is divided into subclusters according to different randomly generated rules. This algorithm assumes that the anomalous data will be isolated in individual clusters in the upper branches of the tree, since they do not comply with the general characteristics of the rest of the data, and therefore tend to split earlier as they do not comply with the general rules created randomly. Another technique used within hierarchical clustering is the so-called Growing Hierarchical Self Organizing Map (GHSOM) [119]. This technique corresponds to an artificial neural network with a hierarchical structure in which each branch makes use of a self-organizing map (SOM) [120].

On the other hand, random forests have also been used for clustering [121]. Although this technique is normally used as a classifier or selection of relevant features, it can also be used for clustering. The algorithm estimates the distance between data based on the number of times they end up on the same leaf. To obtain the clusters, it is necessary to prune the tree by the number of branches corresponding to the number of clusters, so it is necessary a priori to know the number of clusters to be defined.

Another aspect of clustering methods is partition-based clustering. These methods group data into different partitions with no hierarchical relationship between them. Thus, these techniques are based on the assumption that all data belong to normal classes. As soon as a new data appears, it can be considered anomalous if it does not belong to any of the previously defined clusters. Under this assumption, the most widely used technique is K-Means [92, 122–124]. This technique seeks to minimize the sum of distances between the points of a cluster and the cluster centroid. These distances, being measured as average distances between the points to the centroid, are sensitive to the occurrence of anomalies in the data, so other works make use of K-Medoids [125]. This technique is similar to the previous one, making use of the median of the points instead of the mean. The main problem with these techniques is that they require the number of clusters to be formed to be passed as an initialization parameter. This, a priori, is difficult to know, unless you have extensive knowledge of the database you are working with.

On the other hand, the previous techniques present problems when dealing with clusters of different size and density. For this reason, other techniques take this factor into account when detecting anomalies. Thus, one of the algorithms used takes into account the density of a point with respect to that of its neighbors. If this point has a lower density, it is assumed that this is because it corresponds to an anomaly. In particular, the Local Outlier Factor (LOF) technique makes use of this system [117, 126]. On the other hand, following the same principle, the Density-based spatial clustering of applications with noise (DBSCAN) algorithm classifies the data into clusters based on the proper density of the data [127, 128]. However, this method is dependent on the specified density parameter, and therefore does not perform well on databases with different densities.

Beyond the clustering methods, there is a set of techniques that are based on the assumption that the anomalous points are far away from the rest of the normal data. The main difference is that while the previous techniques seek a clustering of the data seeking to minimize the variability between them, these new techniques only focus on the distance between the data to define whether a new sample is anomalous or not. If a data is not close to the rest, it is considered anomalous. One of the techniques used in this sense consists in using the K nearest neighbor technique [129]. In this way, the sum of the distances of the K nearest neighbors is calculated and by means of a threshold it is determined whether it falls within normality or not. Another approach consists of determining the number of neighbors within a given distance range and, likewise, a threshold is used to determine whether it is within normality. The main problem with these methods is their high computational cost, since the distance has to be calculated with respect to the rest of the points in the training database. In addition, these methods do not allow the determination of the different groupings that characterize the database.

However, distance-based techniques can suffer from the Hughes effect in high dimensionality databases [130]. With increasing dimensionality the average distance of the data increases and the variability of the data decreases exponentially. For this reason, some authors prefer to take into account the value of the angles formed by the points to determine whether a point is anomalous or not. The ABOD method [131, 132] considers a point to be anomalous if the variability of the angles of this point with the neighbors is low (considering in this way that they are far from the normal samples), while it will consider it normal, if this point has a high variability of angles.

Within the field of unsupervised techniques, solutions based on deep learning techniques have also been developed. Among these techniques, on the one hand Deep Belief Networks (DBN) are used [133]. On the other hand, Generative Adversarial Networks (GAN) are used for anomaly detection in images [134, 135]. Generative adversarial networks are composed of two neural networks, one discriminative and one generative that compete with each other. The former seeks to detect whether an image is real or generated, while the latter seeks to generate realistic images. GANs learn to generate images within normality. As soon as any modification is introduced as input data, the generative network is not able to generate a coherent image, evidencing the anomaly. Finally, the use of autoencoders has also been highly studied [136–138]. The autoencoders are trained in such a way that they have to reconstruct the input data, having to perform a previous dimensionality reduction. As with GANs, as soon as an anomaly is detected, the trained network is not able to reconstruct it.

Based on the same idea as autoencoders, dimensionality reduction techniques are used for anomaly detection. Dimensionality reduction techniques are based on the assumption that the data can be reduced to a lower dimensional subspace where the difference between normal and anomalous classes is more evident. Thus, among these techniques, the use of Principal Component Analysis (PCA) [95] stands out. This technique, like autoencoders, is based on the idea of reducing the dimensionality of the initial database and detecting anomalies through subsequent reconstruction. One of the major limitations is that only numerical data can be used, after normalization. This technique, in addition to being used on its own, is widely used in the literature as a previous phase of feature extraction [115].

In general, unsupervised techniques are an advantage when it comes to anomaly detection, understood as a change in a normal pattern. Among the main advantages of these techniques is the fact that no prior labeling of the data is required. In this way, the techniques themselves are responsible for characterizing normality in order to subsequently detect those data that deviate from it.

In conclusion, the use of anomaly detection techniques, which have given such good results in other areas and are summarized in Table 3, can be used to detect changes in sitting postural patterns with an individualized approach. Within this approach, the use of unsupervised techniques stands out above the rest, among other factors, because it is not necessary to carry out a prior labeling of the database. However, this is a totally new approach and has not been explored in the literature but of great potential. It is therefore necessary to analyze which of the unsupervised techniques offers the best results, based on quantitative metrics such as percentage of success or computational cost, or qualitative metrics such as model learning capacity or robustness to different normal sitting patterns. This is why it is a new line of research to be considered in the near future to perform postural diagnosis in wheelchair users.

**Table 3:** Summary table of techniques with the new approach

Techniques	Advantages	Limitations	Refs.
Supervised	High success rates Interpretation of results	Limited number of anomalous samples: Unbalanced data base Health specialist presence required for data labeling Inability to characterize all anomalies	[34, 93–102]
Semi- Supervised	Normal samples available Ability to detect unknown anomalies	Since the postural pattern may be composed of different normal states, the normal boundary is wide Expert knowledge required in case of wanting to label different normal states	[19, 67, 103–114]
Unsupervised	No data labeling required Detection of unknown anomalies Applicable to large data sets	Increased tendency to false positives Lack of interpretation Normal data are grouped in clusters assumption	[92, 117–119, 122, 123, 125–127, 129–137]
Unsupervised	No data labeling required Detection of unknown anomalies Applicable to large data sets	Increased tendency to false positives Lack of interpretation Normal data are grouped in clusters assumption	[92, 117–120, 122–127, 129–138]

## Discussion

In the previous chapters, monitoring systems (section Sitting posture monitoring systems) and techniques for detecting anomalies, both from a traditional generalized approach where all postures other than the correct one are considered anomalies (section Traditional approach through generalized techniques), as well as from the newly individual proposed approach (section New and individualized anomaly detection approach), have been presented. During this section their applicability in the field of sitting in people with low mobility is discussed analyzing strengths and areas for improvement to be studied in future research work.

### Discussion on sitting posture monitoring systems

To develop an intelligent, non-intrusive and easy-to-use device for the wheelchair-using population it is necessary to measure and obtain the appropriate variables that will be subsequently be further processed to diagnose the patient’s functional status. There are different types of monitoring systems depending on the location where the sensors are placed, each of them with their advantages and limitations.

The main advantage of the systems located in the environment is that they are not intrusive, as well as object detection capability and fast information processing. This is an indispensable requirement in a sensitive population such as wheelchair users, which this type of system fulfills perfectly. This is achieved by placing sensor elements at fixed points in the environment. Likewise, this type of system allows the simultaneous monitoring of more than one person as long as they are within the range of the cameras.

As a counterpart, this type of system presents a limitation in terms of portability. This is a critical point to take into account, since the aim is to carry out continuous monitoring, so the developed system must be able to move with the user, not limiting itself to monitoring in a limited space. Thus, for example, there is no point in monitoring a patient who has suffered a stroke if it is limited to a closed environment. However, to date, the work developed using vision systems has focused on monitoring users in closed environments with controlled lighting conditions. In addition, not only the information necessary to carry out the postural study is collected, but also perform a continuous monitoring of the life of the people being studied. This can generate privacy problems. Thus, not only their postural state is monitored, but also additional information irrelevant to the application for which they were designed.

Given these limitations of the systems located in the environment, other works are more in favour of using wearable sensors placed on the user. These systems can cope with the greater limitation of the sensors located in the environment, allowing continuous monitoring as they are incorporated on the user himself. Wearable sensors allow information to be collected continuously in a simple and low-cost way, but they can be a nuisance for users. In addition, this type of sensor is highly dependent on the position in which it is placed, so it is necessary to have qualified healthcare personnel to place it. On the other hand, wearable sensors have a tendency to suffer disturbances due to rubbing against the clothing or body of the wheelchair users on which they are placed, which is an additional cause of discomfort in this population. It must be taken into account that some patients, such as stroke patients, will have part of their body paralyzed, so they will not be aware of this friction between the sensor and the body. It is also possible that injuries may occur due to friction. Finally, although they can be of great use in conjunction with other types of sensors, they present difficulties in carrying out correct monitoring on their own. For this reason, the search for other types of monitoring technologies that eliminate these limitations has been intensified.

In this way, finally, the possibility of reaching an intermediate point is sought, using sensors that are sufficiently portable to accompany the user continuously, but at the same time are not intrusive. To this end, the possibility of placing the sensor system in the assistive devices has been considered. In the case of a person with low mobility, in the wheelchair itself. Thus, the use of systems based on force sensors or pressure sensors stands out above the rest, which can be differentiated in two ways: on the one hand, the use of pressure mats composed of a large number of sensors, and on the other hand, the use of sensors located in discrete positions.

While the main advantage of the use of the pressure mats is that it is easy to implement as it is already commercially available, they have certain disadvantages, which must be addressed, including their high price and limited time of use. In addition, since they are a closed solution, there is little room for improvement, both in terms of battery life and data storage for subsequent use of intelligent diagnostic techniques. In the same way, since most of these sensors are not designed for monitoring applications for wheelchair users, it is necessary to adapt these devices to safe environments for use by this group. On the other hand, the use of sensors in an discrete manner can address these limitations, but requires precise placement of the sensors so that the user can be effectively monitored. In addition, further analysis of the implementation of these sensors on different wheelchair user cushions needs to be carried out. It is therefore necessary to continue working on the line of pressure or force sensors, adapting them for use by people with low mobility, both in nursing homes and in their own homes.

### Discussion of anomaly detection techniques for wheelchair users

Having discussed the most necessary points to be addressed in terms of postural monitoring devices, it is necessary to analyze the techniques used to carry out a sitting posture anomaly detection of wheelchair users.

Firstly, it should be noted that the generalized techniques are mainly oriented to a population different from people with low mobility. For this reason, there is a lack of works developed in clinical settings as well as an approach from health perspective to detect posture anomalies. Thus, this paper aims to classify and organize the existing works, with an approach oriented to wheelchair users, thus identifying strengths and limitations of these techniques for this group.

As has been shown above, the use of techniques based on rules or statistics is based on the fact that knowledge of the database is available in order to be able to make use of techniques based on assumptions. However, although the study of postural identification is extensive in office workers, the study in wheelchair users with their particular characteristics is scarce. This means that the assumptions under which these techniques work are not necessarily valid in this population and should be revised. Thus, as an example, these works take into account postures such as leg crossing, which a wheelchair user is unlikely to perform. Generally, the postures of this population, such as patients who have suffered a stroke, will be more related to weakness in the trunk, which will cause lateral or frontal movements on the seat. In the same way, many of these works are not aimed at postural diagnosis in order to carry out an analysis of the functional status of the monitored user. In fact, postural status is used as a tool to check other aspects such as degree of attention at work or stress level. Thus, among the work aimed at wheelchair users, the use of intelligent techniques capable of detecting the intrinsic characteristics of the postural state of this population stands out.

Nevertheless, intelligent techniques have as a point of improvement the fact that they are effective on “familiar” subjects, i.e., subjects whose characteristics are similar to the data used in training. Nevertheless, the effectiveness of the developed intelligent classifiers decreases when validated with data from subjects with different physical characteristics than those used to train the model. Thus, many of these classification models are trained on healthy subjects with healthy physical and muscular builds, in part because of the difficulty of collecting data in a vulnerable population such as wheelchair users. However, these works do not validate the results in the specific population or an analysis is not carried out to ensure that the results obtained are valid for subjects with physical complexions different from those used for training. Despite attempts to select a group of subjects for training that is as heterogeneous as possible, sufficient generalization is not achieved. This is an improvement point to consider, as the wheelchair user population is generally diverse in physical characteristics, primarily due to the different pathologies they experience. In this way, the physical complexion of a person who has suffered a stroke but still retains a certain degree of movement will not be the same as that of a quadriplegic person with absolute immobility. Therefore, the proposed new individual approach may be beneficial in solving this problem.

As seen, approaching the problem with a new perspective, where anomalies are treated as changes in a sitting postural pattern rather than simply as incorrect postures, can be beneficial for detecting changes in the functional state of patients. However, since there are few existing studies in the literature developed under this approach, it is necessary to study the applicability of works developed in other domains when performing anomaly detection in sitting posture. As previously mentioned, within this approach there are three types of techniques: supervised, semi-supervised and unsupervised.

On one hand, in the case of using supervised techniques, it has been observed that, generally, the number of normal samples exceeds the number of anomalous samples, resulting in an imbalanced database for training. While collecting data on their normal postural state pattern might be feasible, gathering a large number of postural states that would characterize their anomalous states would be unfeasible. This imbalance favors the detection of normal classes over anomalous classes. Thus, if you have a person whose natural state in seated position is the thoracic inclination, a large number of samples will be available with respect to this particular postural state. Training a model in a supervised manner also requires the availability of abnormal states such as pelvic tilt, hyperkyphosis or others, which the subject will not perform on his own unless forced.

On the other hand, labeling these samples requires the presence of an specialist in the field. In the case of postural anomaly detection, a healthcare specialist would be needed to characterize the normal sitting posture pattern and all possible postural anomalies that could occur. In the long run, this is impractical for two reasons. Firstly, because a person with limited mobility cannot be required to adopt different postures for the generation of a balanced database as their own pathology makes it impossible for them to adopt a wide range of movements. In this way, and continuing with the example of the stroke patient, if he has lateral paralysis that will lead him to lean to one side, it will be impossible for him to develop postures on the paralyzed side. It would be necessary to expose this subject to a large number of forced postural changes in order to collect anomalous data. Over time, this affects both the user, whose daily life is disturbed, and the healthcare specialist, who must supervise the collection of this data. Secondly, labeling the different postural states, both normal and anomalous, requires the presence of a medical specialist, adding additional workload to healthcare professionals when the ultimate goal of the intelligent system should be to assist them.

The use of semi-supervised techniques can help eliminate the issue of having an imbalanced database, as mentioned, since the system is trained only with labeled data of normality. However, the normal sitting postural pattern can be composed of different postural states, each different from the others. For example, a normal sitting pattern may be characterized by a neutral sitting position, a thoracic rotation and a thoracic rotation in conjunction with a pelvic rotation. Thus, one-class techniques are not applicable since the normality characterized may be too wide, making the boundary defined as normal too wide. So for example, in this case above, a pelvic rotation (without the thoracic) would be considered as an anomaly, but given the similarity with the postural states defined as normal, it could fall within this boundary of normality, resulting in an incorrect outcome. In the case of using multiclass techniques, a healthcare specialist is needed to differentiate between all normal postural states. Like in the previous case, this adds additional workload to healthcare professionals.

Therefore, in general, unsupervised methods constitute an advantage when selected as an anomaly detection technique, as they do not require prior labeling of the data. This fact makes the method more robust in the face of the emergence of new postural states not considered in the database. Thus, even though a significant portion of a person’s postural states may be considered, particular circumstances of each user can be taken into account when characterizing normality. Continuing with the same example as above, a person with a stroke will see part of his or her body paralyzed. This paralysis will vary for each patient, depending on the location and severity of the stroke. This fact makes data labeling more difficult as each user will have a characteristic sitting pattern, so the use of unsupervised techniques could be beneficial in this context. On the other hand, in the case of supervised or semi-supervised techniques, it is necessary to label the data, so that each of the normalities is known to which postural state it corresponds (neutral sitting, thoracic inclinations, pelvic rotations, etc.). However, when working with unsupervised techniques, the medical sense of the data is lost. Similar data is grouped into clusters, but it is unknown to which postural state these clusters correspond. However, this could be addressed by showing the healthcare specialist a sample from each cluster. Since it can be assumed that all clusters will be formed by similar postural states, they only need to label N clusters, not the entire dataset, reducing their workload.

## Conclusions

Having analyzed the main trends related to intelligent postural diagnosis systems, throughout this section the main conclusions obtained are drawn.

The number of individuals requiring the use of a mobility assistance device is on the rise, attributed to the characteristic bone and muscle weakening associated with aging, as well as various neurodegenerative diseases. Monitoring the posture of these individuals and detecting potential anomalous behaviors is essential for conducting a proper functional diagnosis. This allows to carry out a rehabilitation adapted to the situation of each patient, being able to prevent dangerous situations such as ulcers or falls as well as to prevent musculoskeletal problems. All this has an impact on the patient, increasing their quality of life. Throughout this article, an analysis of both devices and techniques for detecting postural anomalies has been carried out, identifying the advantages and limitations of each method. In this way, an orderly and organized understanding of the postural diagnosis topic is provided, offering researchers a resource on which to base future investigations.

In this work, a classification of postural monitoring devices based on their location has been proposed. Thus, they are classified into systems located in the environment, systems located on the user, and systems located on the assistive device. The latter, mostly composed of pressure sensors discretely located, stand out above the rest as they allow for non-intrusive monitoring, maintaining the portability of the monitoring device. However, for proper monitoring it is necessary to determine the exact position of these sensors, so further research is needed in this area.

With these devices, anomaly detection techniques have been applied for functional diagnosis. Traditionally, an approach has been adopted where any posture other than the correct one is considered an anomaly. Thus, the use of supervised techniques is prevalent, extensively employing classifiers based on various methods: rule-based techniques, statistical techniques, and intelligent techniques. While the first two are simpler, they yield poorer results when monitoring a larger number of postures, exceeding 8. Intelligent techniques, on the other hand, have proven to be more effective, albeit at the cost of losing interpretability in the model’s decision-making process.

However, these classifiers based on supervised techniques do not take into account the characteristic postural pattern of each individual. Given the importance of detecting changes in relation to a normal sitting pattern that can indicate a change in the functional state of patients, a new approach is necessary. Classifiers only allow for the detection of incorrect postures. Nonetheless, in the proposed new approach, the sitting postural pattern is characterized individually for each user, and changes from this pattern are detected. Anomaly detection techniques can be categorized as supervised, semi-supervised, and unsupervised. Unsupervised techniques are the most widely used in other domains and hold greater potential in the field of anomaly detection in sitting posture, due, among other factors, to their ability to detect previously unknown anomalies, as well as not requiring a labeled database.

This is a relatively unexplored approach in the field of sitting posture, offering significant opportunities to support healthcare specialists. Therefore, a thorough analysis of the application of successfully used anomaly detection techniques in other domains is necessary when applied to the sitting posture of users with limited mobility. This approach can detect not only isolated anomalous behaviors but also sequential ones.

Thus, and in view of what has been explained throughout this article, it is to be expected that future technological advances will be in line with the two aspects described here: postural monitoring and anomaly detection.

Within the field of monitoring, it is to be expected that technological devices will allow adaptation to the seating cushion of each user. In this way, a thorough analysis must be carried out to determine not only the ideal location of the pressure sensors (the most suitable as we have seen in section Sitting posture monitoring systems), but also to improve their implementation along the wheelchair user’s own sitting cushions. As previously mentioned, pressure or force sensors require rigid surfaces for proper operation. However, the postural cushions of each user are composed of different materials (gel or air as an example), making it difficult to implement the sensors on them. In this way, the postural monitoring device will adapt to the user, and not the other way around. Currently, works have focused on healthy people, but it is to be expected that as future lines of research tend to analyze wheelchair users, research will not only focus on monitoring and detection of postural anomalies, but will also allow detection of anomalies in the course of their daily life, due to external agents among others.

On the other hand, within intelligent anomaly detection systems, future lines of research will tend to further explore anomaly detection methods using an individualized approach. Firstly, since this is a completely new approach in the field of sitting posture anomaly detection, the near future will consist in the study of the methodology and techniques that best suit the issues related to the detection of postural anomalies. Therefore, it is crucial to consider parameters such as the effectiveness of the methodology employed, regardless of the postural monitoring device, the implementation of technology in embedded systems, and the scalability of the systems in the face of new postural anomalies. Finally, within the medical field, works will focus on the direct implementation of the devices in patients, both in their own homes and in clinics.

## Data Availability

Data sharing is not applicable to this article as no datasets were generated or analysed during the current study.

## Abbreviations

CNN: Convolutional Neural Network
DBN: Deep Belief Network
DBSCAN: Density-Based Spatial Clustering of Applications with Noise
DT: Decision Tree
FSR: Force Sensing Resistor
GAN: Generative Adversarial Networks
GHSOM: Growing Hierarchical Self Organizing Map
HMM: Hidden Markov Model
IF: Isolation Forest
IMU: Inertial Measurement Unit
KNN: K-Nearest Neighbor
LOF: Local Outlier Factor
LSTM: Long Short-Term Memory
MLP: Multilayer Perceptron
PCA: Principal Component Analysis
SOM: Self Organizing Map
SVM: Support Vector Machines

## References

[1] Parry S, Chow Marilyn, Batchelor F, Fary RE (2019). Physical activity and sedentary behaviour in a residential aged care facility. Australas J Ageing.

[2] Wullems JA, Verschueren SMP, Degens H, Morse CI, Onambélé GL (2016). A review of the assessment and prevalence of sedentarism in older adults, its physiology/health impact and non-exercise mobility counter-measures. Biogerontology.

[3] Selph SS, Skelly AC, Wasson Ngoc, Dettori JR, Brodt ED, Ensrud E, Elliot D, Dissinger KM, McDonagh M (2021). Physical activity and the health of wheelchair users: a systematic review in multiple sclerosis, cerebral palsy, and spinal cord injury. Arch Phys Med Rehabil.

[4] Tremblay MS, Colley RC, Saunders TJ, Healy GN, Owen N (2010). Physiological and health implications of a sedentary lifestyle. Appl Physiol Nutr Metab.

[5] Zhang Y, Markovic S, Sapir I, Wagenaar RC, Little TDC. Continuous functional activity monitoring based on wearable tri-axial accelerometer and gyroscope. 2011 5th International Conference on Pervasive Computing Technologies for Healthcare and Workshops, PervasiveHealth 2011. 2011;370–373.

[6] van Nes IJW, Nienhuis Bart, Latour Hilde, Geurts Alexander CH (2008). Posturographic assessment of sitting balance recovery in the subacute phase of stroke. Gait Posture.

[7] Mock Michael, Sweeting K. Gait and posture-assessment in general practice. Australian family physician. 2007;36(6). 17565395

[8] Chien C-W, Lin J-H, Wang C-H, Hsueh I-P, Sheu C-F, Hsieh C-L (2007). Developing a short form of the postural assessment scale for people with stroke. Neurorehabil Neural Repair.

[9] Reuter B, Gumbinger C, Sauer T, Wiethölter H, Bruder I, Diehm C, Ringleb PA, Hacke W, Hennerici MG, Kern R (2016). Access, timing and frequency of very early stroke rehabilitation-insights from the baden-wuerttemberg stroke registry. BMC Neurol.

[10] Spilsbury K, Nelson A, Cullum N, Iglesias C, Nixon J, Mason S (2007). Pressure ulcers and their treatment and effects on quality of life: hospital inpatient perspectives. J Adv Nurs.

[11] Sung J, Trace Y, Peterson EW, Sosnoff JJ, Rice LA (2019). Falls among full-time wheelchair users with spinal cord injury and multiple sclerosis: a comparison of characteristics of fallers and circumstances of falls. Disabil Rehabil.

[12] Castro-Avila AC, Seron P, Fan E, Gaete M, Mickan S (2015). Effect of early rehabilitation during intensive care unit stay on functional status: systematic review and meta-analysis. PLoS ONE.

[13] Liu B, Li Y, Zhang S, Ye X (2017). Healthy human sitting posture estimation in RGB-D scenes using object context. Multimedia Tools Appl.

[14] Zaltieri M, Presti DL, Bravi M, Caponero MA, Sterzi S, Schena E, Massaroni C (2023). Assessment of a multi-sensor FBG-based wearable system in sitting postures recognition and respiratory rate evaluation of office workers. IEEE Trans Biomed Eng.

[15] Tsai M-C, Chu ET-H, Lee C-R (2023). An automated sitting posture recognition system utilizing pressure sensors. Sensors.

[16] Jayasinghe U, Janko B, Hwang F, Harwin WS (2023). Classification of static postures with wearable sensors mounted on loose clothing. Sci Rep.

[17] Liu G, Li X, Xu C, Ma L, Li H. FMCW radar-based human sitting posture detection. IEEE Access. 2023;1–1.

[18] Arshad J, Ashraf MA, Asim HM, Rasool N, Jaffery MH, Bhatti SI (2023). Multi-mode electric wheelchair with health monitoring and posture detection using machine learning techniques. Electronics.

[19] Pereira L, da Plácido SH (2023). A novel smart chair system for posture classification and invisible ECG monitoring. Sensors.

[20] Ma C, Li W, Gravina R, Juan D, Li Q, Fortino G (2020). Smart cushion-based activity recognition: prompting users to maintain a healthy seated posture. IEEE Syst Man Cybern Mag.

[21] Muppavram S, Patel N, Nadeem M. Posture Alert. 2018 IEEE Region 10 Symposium, Tensymp 2018. 2019;213–218.

[22] Abobakr A, Hossny M, Nahavandi S (2018). A skeleton-free fall detection system from depth images using random decision forest. IEEE Syst J.

[23] Matar G, Lina JM, Kaddoum G (2020). Artificial neural network for in-bed posture classification using bed-sheet pressure sensors. IEEE J Biomed Health Inform.

[24] Stone EE, Skubic M (2015). Fall detection in homes of older adults using the microsoft kinect. IEEE J Biomed Health Inform.

[25] Lee CC, Saidy L (2019). Human activity recognition based on smart chair. Sensors Mater.

[26] Zemp R, Fliesser M, Wippert PM, Taylor WR, Lorenzetti S (2016). Occupational sitting behaviour and its relationship with back pain - a pilot study. Appl Ergon.

[27] Jiang Y, An J, Liang F, Zuo G, Yi J, Ning C, Zhang H, Dong K, Wang ZL (2022). Knitted self-powered sensing textiles for machine learning-assisted sitting posture monitoring and correction. Nano Res.

[28] Bourahmoune K, Ishac K, Amagasa T (2022). Intelligent posture training: machine-learning-powered human sitting posture recognition based on a pressure-sensing IoT cushion. Sensors.

[29] Roh J, Park HJ, Lee KJ, Hyeong J, Kim S, Lee B (2018). Sitting posture monitoring system based on a low-cost load cell using machine learning. Sensors (Switzerland).

[30] Martins L, Ribeiro B, Almeida R, Pereira H, Jesus A, Quaresma C, Vieira P. Optimization of sitting posture classification based on anthropometric data. HEALTHINF 2016 - 9th International Conference on Health Informatics, Proceedings; Part of 9th International Joint Conference on Biomedical Engineering Systems and Technologies, BIOSTEC 2016. 2016;5(Biostec):406–413.

[31] Zhao M, Beurier G, Wang H, Wang X (2021). Exploration of driver posture monitoring using pressure sensors with lower resolution. Sensors.

[32] Wan Q, Zhao H, Li J, Peng X (2021). Hip positioning and sitting posture recognition based on human sitting pressure image. Sensors (Switzerland).

[33] Zhang X, Fan J, Peng T, Zheng P, Lee CKM, Tang R (2022). A privacy-preserving and unobtrusive sitting posture recognition system via pressure array sensor and infrared array sensor for office workers. Adv Eng Inform.

[34] Ma C, Lee CKM, Juan D, Li Q, Gravina R (2022). Work engagement recognition in smart office. Procedia Comput Sci.

[35] Kuang Y, Guo M, Peng Y, Pei Z (2019). Learner posture recognition via a fusing model based on improved SILTP and LDP. Multimedia Tools Appl.

[36] Zaletelj J, Košir A. Predicting students’ attention in the classroom from Kinect facial and body features. In Eurasip Journal on Image and Video Processing, volume 2017. EURASIP Journal on Image and Video Processing, 2017.

[37] González-Cely AX, Diaz CA, Callejas-Cuervo M, Bastos-Filho T (2023). Optical fiber sensors for posture monitoring, ulcer detection and control in a wheelchair: a state-of-the-art. Disabil Rehabil Assist Technol.

[38] Camboim B, da Rosa Tavares JE, Tavares MC, Barbosa JL (2023). Posture monitoring in healthcare: a systematic mapping study and taxonomy. Med Biol Eng Comput.

[39] Mallare JC, Pineda DF, Trinidad GM, Serafica RD, Villanueva JB, Dela CA, Vicerra RR, Serrano KK, Roxas E. Sitting posture assessment using computer vision. HNICEM 2017 - 9th International Conference on Humanoid, Nanotechnology, Information Technology, Communication and Control, Environment and Management. 2018;pages 1–5.

[40] Yang X, Shen Y. Sitting posture correction device based on infrared distance measurement. 2018 IEEE International Conference on Real-Time Computing and Robotics, RCAR 2018. 2019;607–611.

[41] Saenz-De-Urturi Z, Garcia-Zapirain SB (2016). Kinect-based virtual game for the elderly that detects incorrect body postures in real time. Sensors.

[42] Klishkovskaia T, Aksenov A, Sinitca A, Zamansky A, Markelov O, Kaplun D (2020). Development of classification algorithms for the detection of postures using non-marker-based motion capture systems. Appl Sci.

[43] Walsh P, Dunne LE, Caulfield B, Smyth B (2006). Marker-based monitoring of seated spinal posture using a calibrated single-variable threshold model. Ann Int Confer IEEE Eng Med Biol Proc.

[44] Haghi M, Ershadi A, Deserno T (2023). Recognizing human activity of daily living using a flexible wearable for 3D spine pose tracking. Sensors.

[45] Tang HY, Tan SH, Su TY, Chiang CJ, Chen HH (2021). Upper body posture recognition using inertial sensors and recurrent neural networks. Appl Sci.

[46] Jun D, Johnston V, McPhail SM, O’Leary S (2019). Are measures of postural behavior using motion sensors in seated office workers reliable?. Hum Factors.

[47] Tlili F, Haddad R, Bouallegue R, Shubair R (2022). Design and architecture of smart belt for real time posture monitoring. Internet of Things.

[48] Cristina Ana, Geraldo Ferreira, Kuasne Angela Maria (2020). Prototype of wearable technology applied to the monitoring of the vertebral column. Int J Online Biomed Eng.

[49] Petropoulos A, Sikeridis D, Antonakopoulos T. SPoMo: IMU-based real-Time sitting posture monitoring. IEEE International Conference on Consumer Electronics - Berlin, ICCE-Berlin. 2017;Sept.:5–9.

[50] Benocci M, Farella E, Benini L. A context-aware smart seat. Proceedings of the 4th IEEE International Workshop on Advances in Sensors and Interfaces, IWASI 2011. 2011;104–109.

[51] Awais M, Raza M, Ali K, Ali Z, Irfan M, Chughtai O, Khan I, Kim S, Rehman MU (2019). An internet of things based bed-egress alerting paradigm using wearable sensors in elderly care environment. Sensors.

[52] Bei S, Xing Z, Taocheng L, Qin L. Sitting posture detection using adaptively fused 3D features. Proceedings of the 2017 IEEE 2nd Information Technology, Networking, Electronic and Automation Control Conference, ITNEC 2017. 2018;2018-January:1073–1077.

[53] Pierleoni P, Belli A, Maurizi L, Palma L, Pernini L, Paniccia M, Valenti S (2016). A wearable fall detector for elderly people based on AHRS and barometric sensor. IEEE Sens J.

[54] Chopra S, Kumar M, Sood S. Wearable posture detection and alert system. Proceedings of the 5th International Conference on System Modeling and Advancement in Research Trends, SMART 2016. 2017;130–134.

[55] El-Sayed B, Farra N, Moacdieh N, Hajj H, Haidar R, Hajj Z. A novel mobile wireless sensing system for realtime monitoring of posture and spine stress. 2011 1st Middle East Conference on Biomedical Engineering, MECBME 2011. 2011;428–431.

[56] Qian Z, Bowden AE, Zhang D, Wan J, Liu W, Li X, Baradoy D, Fullwood DT (2018). Inverse piezoresistive nanocomposite sensors for identifying human sitting posture. Sensors.

[57] Shi J, Wang L, Dai Z, Zhao L, Mingde D, Li H, Fang Y (2018). Multiscale hierarchical design of a flexible piezoresistive pressure sensor with high sensitivity and wide linearity range. Small.

[58] Ribeiro B, Pereira H, Almeida R, Ferreira A, Martins L, Quaresma C, Vieira P. Optimization of sitting posture classification based on user identification. Proceedings. IEEE 4th Portuguese Meeting on Bioengineering. ENBENG. 2015;2015:2015.

[59] Kim M, Kim H, Park J, Jee KK, Lim JA, Park MC (2018). Real-time sitting posture correction system based on highly durable and washable electronic textile pressure sensors. Sens Actuators, A.

[60] Yang Y, Wang J, Gao Z, Zhou Y. Design and preliminary evaluation of an air-alternating wheelchair seating system for pressure ulcer prevention. ICBBT 2010 - 2010 International Conference on Bioinformatics and Biomedical Technology. 2010;239–243.

[61] Arnrich B, Setz C, La Marca R, Tröster G, Ehlert U (2010). What does your chair know about your stress level?. IEEE Trans Inf Technol Biomed.

[62] Arias DE, Pino EJ, Aqueveque P, Curtis DW. Unobtrusive support system for prevention of dangerous health conditions in wheelchair users. Mobile Inf Syst. 2016;2016.

[63] Fard FD, Moghimi S, Lotfi R (2013). Evaluating pressure ulcer development in wheelchair-bound population using sitting posture identification. Engineering.

[64] Perez N, Vermander P, Lara E, Mancisidor A, Cabanes I. Sitting posture monitoring device for people with low degree of autonomy. In International Conference on NeuroRehabilitation. 2020;pages 305–310. Springer.

[65] Tavares C, Silva J, Mendes A, Rebolo L, Fatima DM, Alberto N, Lima M, Radwan A, Da Silva HP, Antunes P. Smart office chair for working conditions optimization. IEEE Access. 2023;(April):50497–50509.

[66] Luna-Perejón F, Montes-Sánchez JM, Durán-López L, Vazquez-Baeza A, Beasley-Bohórquez I, Sevillano-Ramos JL (2021). Iot device for sitting posture classification using artificial neural networks. Electronics (Switzerland).

[67] Ma C, Li W, Gravina R, Cao J, Li Q, Fortino G (2017). Activity level assessment using a smart cushion for people with a sedentary lifestyle. Sensors.

[68] Vermander P, Mancisidor A, Cabanes I, Perez N, Torres-Unda J (2023). Intelligent sitting posture classifier for wheelchair users. IEEE Trans Neural Syst Rehabil Eng.

[69] Bibbo D, Carli M, Conforto S, Battisti F (2019). A sitting posture monitoring instrument to assess different levels of cognitive engagement. Sensors.

[70] Liang G, Cao J, Liu X. Smart cushion: a practical system for fine-grained sitting posture recognition. In 2017 IEEE International Conference on Pervasive Computing and Communications Workshops, PerCom Workshops 2017. 2017;419–424.

[71] Kumar R, Bayliff A, De D, Evans A, Das S, Makos M. Care-chair: sedentary activities and behavior assessment with smart sensing on chair backrest. 2016 IEEE International Conference on Smart Computing, SMARTCOMP 2016. 2016;1–8.

[72] Jeong H, Park W (2021). Developing and evaluating a mixed sensor smart chair system for real-time posture classification: combining pressure and distance sensors. IEEE J Biomed Health Inform.

[73] La Mura M, De Gregorio M, Lamberti P, Tucci V (2023). Iot system for real-time posture asymmetry detection. Sensors.

[74] Yoon H, Hwang S, Jung D, Choi S, Joo K, Choi J, Lee Y, Do Jeong U, Park K. Estimation of sleep posture using a patch-type accelerometer based device. Proceedings of the Annual International Conference of the IEEE Engineering in Medicine and Biology Society, EMBS. 2015;2015-Novem:4942–4945. 10.1109/EMBC.2015.7319500 26737400

[75] Mesanza AB, Lucas S, Zubizarreta A, Cabanes I, Portillo E, Rodriguez-Larrad A (2020). A machine learning approach to perform physical activity classification using a sensorized crutch tip. IEEE Access.

[76] Nahavandi Darius, Abobakr Ahmed, Haggag Hussein, Hossny Mohammed. A Low Cost Anthropometric Body Scanning System Using Depth Cameras. *Proceedings - 2018 IEEE International Conference on Systems, Man, and Cybernetics, SMC 2018*. 2019;pages 3486–3491.

[77] Liu W, Guo Y, Yang J, Hu Y, Wei D. Sitting posture recognition based on human body pressure and CNN. AIP Conference Proceedings. 2019;2073(February).

[78] Kim W, Jin B, Choo S, Nam CS, Yun MH (2019). Designing of smart chair for monitoring of sitting posture using convolutional neural networks. Data Technol Appl.

[79] Amato F, López A, Peña-Méndez E, Vaňhara P, Hampl Al, Havel J (2013). Artificial neural networks in medical diagnosis. J Appl Biomed.

[80] Oniga S, Suto J (2016). Activity recognition in adaptive assistive systems using artificial neural networks. Elektronika ir Elektrotechnika.

[81] Estrada JE, Vea LA. Real-time human sitting posture detection using mobile devices. In Proceedings - 2016 IEEE Region 10 Symposium, TENSYMP 2016. 2016;140–144.

[82] Fida B, Proto A, Bibbo D, Conforto S, Bernabucci I, Schmid M. Real time event-based segmentation to classify locomotion activities through a single inertial sensor. MOBIHEALTH 2015 - 5th EAI International Conference on Wireless Mobile Communication and Healthcare - Transforming Healthcare through Innovations in Mobile and Wireless Technologies. 2015.

[83] Cheng J, Sundholm M, Zhou B, Hirsch M, Lukowicz P (2016). Smart-surface: large scale textile pressure sensors arrays for activity recognition. Pervasive Mob Comput.

[84] Pazhoumand-Dar H (2019). FAME-ADL: a data-driven fuzzy approach for monitoring the ADLs of elderly people using Kinect depth maps. J Ambient Intell Humaniz Comput.

[85] Ribeiro B, Martins L, Pereira H, Almeida R, Quaresma C, Ferreira A, Vieira P. Sitting posture detection using fuzzy logic development of a neuro-fuzzy algorithm to classify postural transitions in a sitting posture. HEALTHINF 2015 - 8th International Conference on Health Informatics, Proceedings; Part of 8th International Joint Conference on Biomedical Engineering Systems and Technologies, BIOSTEC 2015. 2015;pages 191–199.

[86] Reguera-García MM, Leirós-Rodríguez R, Álvarez-Barrio L, Fradejas BA-C (2020). Analysis of postural control in sitting by pressure mapping in patients with multiple sclerosis, spinal cord injury and friedreich’s ataxia: a case series study. Sensors.

[87] Mohan A, Choksi M, Zaveri M. Anomaly and activity recognition using machine learning approach for video based surveillance. In 2019 10th International Conference on Computing, Communication and Networking Technologies (ICCCNT). 2019; 1–6. IEEE.

[88] Zhang Y, Chen Y, Wang J, Pan Z. Unsupervised deep anomaly detection for multi-sensor time-series signals. IEEE Transactions on Knowledge and Data Engineering. 2021.

[89] Minh HD, Hoang MN, Tung LN, Duc MN, Cong KN, Tran DD. AICARE: Health abnormality detection of elderly automatically using deep learning. 2023;340–345.

[90] Gao X, Chen Z, Tang S, Zhang Y, Li J (2016). Adaptive weighted imbalance learning with application to abnormal activity recognition. Neurocomputing.

[91] Sheikh SM, Ngebani I. A personal health care office chair. 2nd International Conference on Computer Applications and Information Security, ICCAIS 2019. 2019;1–4.

[92] Vermander P, Mancisidor A, Fortino G, Cabanes I, Gravina R. Unsupervised learning-based methodology for detection of postural anomalies in wheelchair users. In IEEE Conference on Systems, Man, and Cybernetics. 2023;IEEE.

[93] Anton SD, Kanoor S, Fraunholz D, Schotten H. Evaluation of machine learning-based anomaly detection algorithms on an industrial modbus/tcp data set. In Proceedings of the 13th international conference on availability, reliability and security. 2018;1–9.

[94] Afrooz P, Amir HG, Mohammad E, An application of anomaly detection algorithm (2014). A data mining approach for fault diagnosis: an application of anomaly detection algorithm. Measurement.

[95] He S, Zhu J, He P, Lyu MR. Experience report: system log analysis for anomaly detection. In 2016 IEEE 27th international symposium on software reliability engineering (ISSRE). 2016; 207–218. IEEE.

[96] Chkirbene Zina, Eltanbouly Sohaila, Bashendy May, AlNaimi Noora, Erbad Aiman. Hybrid machine learning for network anomaly intrusion detection. In *2020 IEEE international conference on informatics, IoT, and enabling technologies (ICIoT)*. 2020;pages 163–170. IEEE.

[97] Salman T, Bhamare D, Erbad A, Jain R, Samaka M. Machine learning for anomaly detection and categorization in multi-cloud environments. In 2017 IEEE 4th international conference on cyber security and cloud computing (CSCloud). 2017; 97–103. IEEE.

[98] D’angelo G, Palmieri F, Ficco M, Rampone S (2015). An uncertainty-managing batch relevance-based approach to network anomaly detection. Appl Soft Comput.

[99] Haider Waqas, Hu Jiankun, Xie Miao. Towards reliable data feature retrieval and decision engine in host-based anomaly detection systems. In 2015 IEEE 10th Conference on Industrial Electronics and Applications (ICIEA). 2015;pages 513–517. IEEE.

[100] Doelitzscher F, Knahl M, Reich C, Clarke N. Anomaly detection in iaas clouds. In 2013 IEEE 5th International Conference on Cloud Computing Technology and Science, volume1. 2013;pages 387–394. IEEE.

[101] Yin C, Zhu Y, Fei J, He X (2017). A deep learning approach for intrusion detection using recurrent neural networks. IEEE Access.

[102] Han S-J, Cho SB. Evolutionary neural networks for anomaly detection based on the behavior of a program. IEEE Trans Syst Man Cybern Part B Cybern. 2006;36(3):559–70. 10.1109/tsmcb.2005.860136 16761810

[103] Erfani SM, Rajasegarar S, Karunasekera S, Leckie C (2016). High-dimensional and large-scale anomaly detection using a linear one-class svm with deep learning. Pattern Recogn.

[104] Tian Y, Mirzabagheri M, Bamakan SMH, Wang H, Qiang Q (2018). Ramp loss one-class support vector machine; a robust and effective approach to anomaly detection problems. Neurocomputing.

[105] Otamendi J, Zubizarreta A, Portillo E. Machine learning-based gait anomaly detection using a sensorized tip: an individualized approach. Neural Comput Appl. 2023;1–17.

[106] Duan G, Lv H, Wang H, Feng G (2022). Application of a dynamic line graph neural network for intrusion detection with semisupervised learning. IEEE Trans Inf Forensics Secur.

[107] Batra R, Mahajan M, Goel A. An optimized active learning tcm-knn algorithm based on intrusion detection system. In Congress on Intelligent Systems: Proceedings of CIS 2021, Volume 1. 2022;621–634. Springer.

[108] Kuang L, Zulkernine M. An anomaly intrusion detection method using the csi-knn algorithm. In Proceedings of the 2008 ACM symposium on Applied computing. 2008; 921–926.

[109] Hussain B, Qinghe D, Ren P (2018). Semi-supervised learning based big data-driven anomaly detection in mobile wireless networks. China Commun.

[110] Khan ZA, Sohn W (2011). Abnormal human activity recognition system based on r-transform and kernel discriminant technique for elderly home care. IEEE Trans Consum Electron.

[111] Pang G, Shen C, vanden Hengel A. Deep anomaly detection with deviation networks. In Proceedings of the 25th ACM SIGKDD international conference on knowledge discovery & data mining. 2019;353–362.

[112] Du M, Li F, Zheng G, Srikumar V. Deeplog: anomaly detection and diagnosis from system logs through deep learning. In Proceedings of the 2017 ACM SIGSAC conference on computer and communications security. 2017;1285–1298.

[113] Alrawashdeh K, Purdy C. Reducing calculation requirements in fpga implementation of deep learning algorithms for online anomaly intrusion detection. In 2017 IEEE National Aerospace and Electronics Conference (NAECON). 2017;57–62. IEEE.

[114] Nanduri A, Sherry L. Anomaly detection in aircraft data using recurrent neural networks (rnn). In 2016 Integrated Communications Navigation and Surveillance (ICNS). 2016;pages 5C2–1. IEEE.

[115] Nassif AB, Talib MA, Nasir Q, Dakalbab FM (2021). Machine learning for anomaly detection: a systematic review. Ieee Access.

[116] Prasad NR, Almanza-Garcia S, Thomas T (2009). Anomaly detection. Comput Mater Continua.

[117] Shriram S, Sivasankar E. Anomaly detection on shuttle data using unsupervised learning techniques. In 2019 International Conference on Computational Intelligence and Knowledge Economy (ICCIKE). 2019;pages 221–225. IEEE.

[118] Vartouni A, Kashi SS, Teshnehlab M. An anomaly detection method to detect web attacks using stacked auto-encoder. In 2018 6th Iranian Joint Congress on Fuzzy and Intelligent Systems (CFIS). 2018;pages 131–134. IEEE.

[119] Ippoliti Dennis, Zhou Xiaobo (2012). A-ghsom: an adaptive growing hierarchical self organizing map for network anomaly detection. J Parallel Distrib Comput.

[120] Olszewski D, Iwanowski M, Graniszewski W (2024). Dimensionality reduction for detection of anomalies in the iot traffic data. Futur Gener Comput Syst.

[121] Yihunie F, Abdelfattah E, Regmi A. Applying machine learning to anomaly-based intrusion detection systems. In 2019 IEEE Long Island Systems, Applications and Technology Conference (LISAT). 2019;1–5.

[122] Parwez MS, Rawat DB, Garuba M (2017). Big data analytics for user-activity analysis and user-anomaly detection in mobile wireless network. IEEE Trans Industr Inf.

[123] Kumari R, Singh Sheetanshu MK, Jha R, Singh NK. Anomaly detection in network traffic using k-mean clustering. In 2016 3rd International Conference on Recent Advances in Information Technology (RAIT). 2016;387–393.

[124] Ali M, Scandurra P, Moretti F, Sherazi HHR. Anomaly detection in public street lighting data using unsupervised clustering. IEEE Transactions on Consumer Electronics. 2024.

[125] Chitrakar R, Chuanhe H. Anomaly detection using support vector machine classification with k-medoids clustering. In 2012 Third Asian Himalayas International Conference on Internet. 2012;1–5.

[126] Punmiya R, Zyabkina O, Choe S, Meyer J. Anomaly detection in power quality measurements using proximity-based unsupervised machine learning techniques. In 2019 Electric Power Quality and Supply Reliability Conference (PQ) & 2019 Symposium on Electrical Engineering and Mechatronics (SEEM). 2019;1–6.

[127] Thang TM, Kim J. The anomaly detection by using dbscan clustering with multiple parameters. In 2011 International Conference on Information Science and Applications. 2011;1–5.

[128] Liu M, Wang T, Zhang Q, Pan C, Liu S, Chen Y, Lin D, Feng S (2024). An outlier removal method based on pca-dbscan for blood-sers data analysis. Anal Methods.

[129] Ting KM, Washio T, Wells JR, Aryal S (2017). Defying the gravity of learning curve: a characteristic of nearest neighbour anomaly detectors. Mach Learn.

[130] Zamora J. Recent advances in high-dimensional clustering for text data. Claudio Moraga: a passion for multi-valued logic and soft computing. 2017;323–337.

[131] Abdelrahman O, Keikhosrokiani P (2020). Assembly line anomaly detection and root cause analysis using machine learning. IEEE Access.

[132] Alimohammadi H, Chen SN (2022). Performance evaluation of outlier detection techniques in production timeseries: a systematic review and meta-analysis. Expert Syst Appl.

[133] Längkvist M, Karlsson L, Loutfi A (2012). Sleep stage classification using unsupervised feature learning. Adv Artif Neural Syst.

[134] Schlegl T, Seeböck P, Waldstein SM, Schmidt-Erfurth U, Langs G. Unsupervised anomaly detection with generative adversarial networks to guide marker discovery. In Information Processing in Medical Imaging: 25th International Conference, IPMI 2017, Boone, NC, USA, June 25–30, 2017, Proceedings. 2017;pages 146–157. Springer.

[135] Deecke L, Vandermeulen R, Ruff L, Mandt S, Kloft M. Image anomaly detection with generative adversarial networks. In Machine Learning and Knowledge Discovery in Databases: European Conference, ECML PKDD 2018, Dublin, Ireland, September 10–14, 2018, Proceedings, Part I 18. 2019;pages 3–17. Springer.

[136] Zhao Y, Deng B, Shen C, Liu Y, Lu H, Hua X-S. Spatio-temporal autoencoder for video anomaly detection. In Proceedings of the 25th ACM international conference on Multimedia. 2017;1933–1941.

[137] Cozzolino D, Verdoliva L. Single-image splicing localization through autoencoder-based anomaly detection. In 2016 IEEE international workshop on information forensics and security (WIFS). 2016;pages 1–6. IEEE.

[138] Xinji Q, Liu Z, Wu CQ, Hou A, Yin X, Chen Z (2024). Mfgan: multimodal fusion for industrial anomaly detection using attention-based autoencoder and generative adversarial network. Sensors.

